# Functionalized Enzyme-Responsive Biomaterials to Model Tissue Stiffening *in vitro*

**DOI:** 10.3389/fbioe.2020.00208

**Published:** 2020-04-08

**Authors:** Annalisa Tirella, Giorgio Mattei, Margherita La Marca, Arti Ahluwalia, Nicola Tirelli

**Affiliations:** ^1^BioEngineered Systems Lab, Division of Pharmacy and Optometry, School of Health Sciences, Faculty of Biology, Medicine and Health, The University of Manchester, Manchester, United Kingdom; ^2^North West Centre of Advanced Drug Delivery (NoWCADD), Division of Pharmacy and Optometry, School of Health Sciences, Faculty of Biology, Medicine and Health, The University of Manchester, Manchester, United Kingdom; ^3^Department of Information Engineering, University of Pisa, Pisa, Italy; ^4^Research Centre “E. Piaggio”, University of Pisa, Pisa, Italy; ^5^Laboratory of Polymers and Biomaterials, Fondazione Istituto Italiano di Tecnologia, Genoa, Italy

**Keywords:** hydrogels, photo-crosslinking, functionalized polymers, mechanical properties, *in vitro* models, bioinks, rheology

## Abstract

The mechanical properties of the cellular microenvironment play a crucial role in modulating cell function, and many pathophysiological processes are accompanied by variations in extracellular matrix (ECM) stiffness. Lysyl oxidase (LOx) is one of the enzymes involved in several ECM-stiffening processes. Here, we engineered poly(ethylene glycol) (PEG)-based hydrogels with controlled mechanical properties in the range typical of soft tissues. These hydrogels were functionalized featuring free primary amines, which allows an additional chemical LOx-responsive behavior with increase in crosslinks and hydrogel elastic modulus, mimicking biological ECM-stiffening mechanisms. Hydrogels with elastic moduli in the range of 0.5–4 kPa were obtained after a first photopolymerization step. The increase in elastic modulus of the functionalized and enzyme-responsive hydrogels was also characterized after the second-step enzymatic reaction, recording an increase in hydrogel stiffness up to 0.5 kPa after incubation with LOx. Finally, hydrogel precursors containing HepG2 (bioinks) were used to form three-dimensional (3D) *in vitro* models to mimic hepatic tissue and test PEG-based hydrogel biocompatibility. Hepatic functional markers were measured up to 7 days of culture, suggesting further use of such 3D models to study cell mechanobiology and response to dynamic variation of hydrogels stiffness. The results show that the functionalized hydrogels presented in this work match the mechanical properties of soft tissues, allow dynamic variations of hydrogel stiffness, and can be used to mimic changes in the microenvironment properties of soft tissues typical of inflammation and pathological changes at early stages (e.g., fibrosis, cancer).

## Introduction

Many *in vitro* models have been developed to mechanistically investigate biological processes. Most commonly, they are two-dimensional (2D, cell cultured as monolayers), and recent studies evidenced how they may lack the actual complexity of tissues and organs (Mattei et al., 2014a; Fitzgerald et al., 2015; Tirella et al., 2015; Melissaridou et al., 2019). Engineered *in vitro* models represent an alternative, where cells are surrounded by an extracellular matrix (ECM) similar to native tissue, which in its natural state is a complex three-dimensional (3D) network of glycosaminoglycans, adhesion proteins, and structural fibers. ECM provides support and signals to regulate cells adhesion, proliferation, differentiation, morphology, and gene expression (Frantz et al., 2010; Humphrey et al., 2014; Theocharis et al., 2016). Over the last decade, many studies have evidenced how the ECM mechanical properties play essential roles in directing cell behavior and function during tissue development, homeostasis, and disease through mechanotransduction processes (Vining and Mooney, 2017). A number of studies have shown the influence of substrate elasticity (or stiffness) on biological processes, for example, by culturing cells on a variety of natural (e.g., collagen, gelatin) and synthetic (e.g., polyacrylamide) hydrogels mimicking the native stiffness of different biological tissues (Engler et al., 2006; Mattei et al., 2015, 2017; Olivares-Navarrete et al., 2017). Of interest, while engineering a biomaterial to model biological tissues, is the control over dynamic variation of the mechanical properties: in fact, several (if not all) biological processes *in vivo* involve a constant remodeling of the surrounding ECM changing physicochemical properties to match the values critical for tissue development and function (Bonnans et al., 2014; Manou et al., 2019). Among human organs, the liver is known to exhibit a marked mechanosensitive behavior, with hepatic cells being sensitive to changes in liver ECM stiffness. This suggests that (1) mechanical cues could provide prefibrotic signals (Carver and Goldsmith, 2013), and (2) matrix stiffening could contribute to disease progression (Karsdal et al., 2015; Zhubanchaliyev et al., 2016).

Engineering dynamic substrates that replicate the variations of biomechanical properties of ECM *in vitro* would be of high interest to better understand cell mechanotransduction, as well as develop strategies for understanding and controlling pathophysiological processes. There are few examples reported in the literature that uses external triggers to control biomaterial variations of stiffness in time and amplitude, with examples of decreasing elastic moduli due to hydrolysis or enzymatic remodeling of biodegradable polymers (Peng et al., 2018) or to exposure of photocleavable hydrogels to light exposure (Kloxin et al., 2010; Yao et al., 2017). Other examples of increasing elastic moduli use more biomimetic approaches incubating poly(ethylene glycol) (PEG)–fibrinogen with thrombin (Kesselman et al., 2013) or after light exposure of modified PEG polymers (Mabry et al., 2015). Similarly, collagen–alginate hydrogels can be stiffened with addition of divalent cations on demand, but with limited stability (Gillette et al., 2010). External stimuli, such as pH (Yoshikawa et al., 2011) and temperature (Yamato et al., 2001), can be used to alter matrix stiffness, but reported to induce changes in hydrophilic/hydrophobic behavior, hence in volume. Guvendiren and Burdick (2012) developed a hyaluronic acid hydrogel that can be stiffened at a user-defined time during cell culture via a UV radical photopolymerization. This approach enables a rapid substrate stiffening on demand, but limitations of this approach could be the potential cytotoxicity and risk of peptide denaturation as results of UV light radiation (Williams et al., 2005; Fedorovich et al., 2009). Alternative approach was proposed by Stowers et al. (2015) in which the elastic modulus of alginate hydrogel-containing liposomes loaded with CaCl_2_ and gold nanorods is increased when irradiation of gold nanorods causes liposomes breakdown, hence CaCl_2_ release. A more sophisticated approach was recently reported by Liu et al. (2018) using a protein–polymer hydrogel biomaterials that can respond to different triggers using calmodulin-based linkers, photosensitive light, oxygen, and voltage sensing domain 2 (LOV2) protein to enable cyclic modulation of the mechanical properties of the cell-laden constructs.

In physiological stiffening processes, crosslinking enzymes such as lysyl oxidase (LOx) and transglutaminase are expressed by cells to remodel and crosslink ECM. We focused on engineering 3D *in vitro* models to mimic pathophysiological states of hepatic tissue. Enzyme-responsive polymers and a mild biocompatible two-step curing scheme was designed to be tolerated by encapsulated cells and a controlled increase of elastic modulus. In this study, we designed hydrogels with functional groups acting as substrates on exposure to such enzymes. Poly(ethylene glycol), a hydrophilic water-soluble polymer, was chosen because of its biocompatibility and potential modularity, allowing decoupling of hydrogel biochemical and mechanical properties (Peyton et al., 2006). Crosslinked PEG hydrogels are not degradable by mammalian enzymes and do not interact with proteins, thus acting as inert matter in physiological environments. It is well known that PEG hydrogels can be functionalized with specific ligands to selectively control cell adhesion and behavior (Hern and Hubbell, 1998; Jonker et al., 2015). In this work, hydrogel precursor solutions composed of different mixtures of monoacrylate and diacrylate PEG derivatives were functionalized with a cysteine-containing RGD peptide selected to act as a cell adhesion motif [*in situ* Michael-type addition prior to crosslinking (Park et al., 2003)], as well as substrate to react in the second- step enzymatic stiffening reaction. Different amounts of primary amines were included in PEG hydrogels to control the stiffening step toward the engineering of diseased *in vitro* models. Poly(ethylene glycol)-based hydrogels were engineered to have a first gelation step using visible light to photochemically crosslink the monomer mixtures, and a second on-demand step (i.e., enzymatic reaction) after incubation with enzymes to form additional crosslinks and further increase hydrogel elastic modulus to mimic *in vivo* processes.

The initial elastic modulus of PEG hydrogels was tuned through the monofunctional/difunctional monomer ratio to match the values of a healthy human liver and being in the range of 0.1–5 kPa. The additional curing step was designed to mimic the fibrotic/stiffening events reported in liver injury: we selected LOx, an enzyme that is up-regulated early in liver injury (Desmoulière et al., 1997; Mesarwi et al., 2015) and contributes to ECM stiffening during liver fibrosis (Perepelyuk et al., 2013; Liu et al., 2016), to crosslink further PEG hydrogels on demand, hence increase the elastic modulus and match early and late events of hepatic tissue fibrosis.

## Materials and Methods

### Hydrogel Design Strategy

The design strategy was based on two major hypotheses: (1) the molar ratio of monoacrylate over diacrylate PEG derivatives in the precursor solution inversely affects the crosslinking density and hence their stiffness after the first (photo-crosslinking) reaction; (2) the stiffening owed to the second (enzymatic) crosslinking reaction is proportional to the amount of tethered free amino groups. Poly(ethylene glycol)-based hydrogels were functionalized and made adhesive to cells by introducing the cysteine-containing integrin-binding peptide GCGYGRGDSPG via Michael-type addition onto PEG diacrylate (PEGDA) acrylates (Lutolf et al., 2001). The same addition strategy was used to tether cysteamine to PEGDA acrylates, thus providing free amines as substrate for the second-step LOx-mediated crosslinking. Density of adhesion sites was determined as 250 μM of RDG-peptide for all the investigated hydrogel formulations, according to published reports (Lutolf et al., 2003; Guvendiren and Burdick, 2012). To tune the initial stiffness, we selected three hydrogel families with different molar ratios between acrylate groups belonging to the monoacrylate PEG-derivatives and those belonging to the diacrylate ones in the hydrogel precursor solution (here coded as A/DA ratios). For all hydrogel formulations, the PEG concentration was fixed to 5% wt/vol, whereas 0.25, 0.5, and 0.75 A/DA ratios were used. From the first design hypothesis, a decrease in initial hydrogel stiffness is expected with increasing A/DA ratio, because monoacrylate PEG derivatives act as pendant chains within the hydrogel network, not contributing to form effective crosslinks. In order to increase the number of crosslinks via the second-step enzymatic crosslinking, we functionalized each A/DA family with three different amounts of amino groups, aiming at hydrogel stiffening to different extents and proportional to the total content of tethered amines. The latter was expressed as the molar ratio between amines and PEGDA acrylate groups prior to the Michael-type addition (i.e., NH_2_/A_PEGDA_ = 0.00, 0.05, and 0.10 mol/mol, where A_PEGDA_ = 2 × moles of initial PEGDA prior the Michael-type addition).

### Materials

Unless otherwise specified, all the materials were purchased from Sigma-Aldrich (Gillingham, United Kingdom). Poly(ethylene glycol) diacrylate (PEGDA, prod. no. 437441) with number average molecular weight ($\bar{M_{\text{n}}}$) of 575 Da and PEG methyl ether acrylate (PEGMA, prod. no. 454990) with $\bar{M_{\text{n}}}$ = 480 Da were purified as follows. Hexane (prod. no. 270504) extraction was used to remove butylated hydroxytoluene radical inhibitor from PEGMA, and then monomethyl ether hydroquinone radical inhibitor was removed from both macromers via elution in dichloromethane through inhibitor removers prepacked columns (prod. no. 306312). Rotary evaporation (Buchi Rotavapor, Flawil, Switzerland) was used to remove the solvent, assessing the efficiency of the process via ^1^H NMR (absence of inhibitor peaks). Purified macromers were stored at −80°C until use. Eosin Y (prod. no. 23′251), 1-vinyl-2-pyrrolidinone (NVP, prod. no. V3409), triethanolamine (TEA, prod. no. 90279), cysteamine hydrochloride (CysAm⋅HCl, prod. no. M6500), lysine oxidase from *Trichoderma viride* (LOx, prod. no. L6150), copper sulfate (prod. no. 451657), L-ascorbic acid (prod. no. A0278), glyoxal solution 40% wt/wt (GlyO, prod. no. 128465), 5,5′-dithiobis(2-nitrobenzoic acid) (DTNB, or Ellman’s reagent, prod. no. D8130), and ninhydrin 2% wt/vol solution (prod. no. N7285) were used as received. GCGYGRGDSPG peptide (>9%) was purchased by Biomatik (Wilmington, DE, United States) and used as received.

### Hydrogel Preparation

Hydrogel precursor solutions with the desired A/DA ratio were prepared in 10 mM phosphate-buffered saline (PBS 1×) independently of the PEGDA functionalization and following the preparative scheme reported in section “Preparation of Hydrogel Precursor Solutions” in Supplementary Material. Formulations investigated in this work are summarized in Table 1. Specifically, each hydrogel is identified by two ratios: (1) A/DA ratio, linked to initial mechanical properties; and (2) NH_2_/A_PEGDA_ ratio, proportional to amine functionalization. The pH of each precursor solution was buffered at value of 7.4, suitable for 3D cell encapsulation. Collagen hydrogels were used as control for cell tests. Type I collagen was extracted from rat tails in sterile conditions following a standard procedure (Beken et al., 1998) and then freeze-dried and dissolved in 0.1% (vol/vol) acetic acid before use to obtain a 3.0 mg/mL collagen solution. The latter was then neutralized on ice by adding Medium 199 10× (M199; Sigma-Aldrich) in a 1:9 volume ratio with 3.0 mg/mL to obtain a 2.7 mg/mL collagen hydrogel precursor and incubated for 30 min (37°C, 5% CO_2_) allowing gelation.

**TABLE 1:** List of components and their concentrations used to prepare the precursor solutions for the investigated hydrogels.

A/DA	0.25			0.50			0.75		
			
NH_2_/A_PEGDA_	0.00	0.05	0.10	0.00	0.05	0.10	0.00	0.05	0.10
PEGDA (mM)	61.57	67.54	74.78	47.62	52.39	58.22	38.84	42.81	47.68
PEGMA (mM)	30.41	23.26	14.58	47.12	41.41	34.43	57.64	52.89	47.05
CysAm (mM)	0.00	6.75	14.96	0.00	5.24	11.64	0.00	4.28	9.54
RGD (mM)	0.25								
Photoinitiating system	0.1 mM eosin Y, 10 mM TEA, 90 mM NVP								

### Rheological Tests

#### Gelation Kinetic

The crosslinking processes were monitored in small-strain oscillatory shear experiments using a Gemini Advanced Rheometer (Bohlin Instruments, Cirencester, United Kingdom). The rheometer was equipped with a 5-mm-thick quartz support (lower plate) coupled to an EXFO Omnicure S1000 lamp (EXFO, Cirencester, United Kingdom) enabling the irradiation of samples during measurements (Ouasti et al., 2011). A parallel plate configuration with a 25-mm-diameter upper plate was used to measure the storage (*G*′) and loss (*G*″) shear moduli over time. Briefly, 50 μL of hydrogel precursor solution was deposited on the quartz bottom plate. Then, the upper plate was lowered down to a gap of 0.1 mm and covered with dodecane to minimize water evaporation during experiments. The samples were irradiated (460 nm, 140 mW/cm^2^, 600 s), whereas *G*′ and *G*″ were recorded up to 900 s, to assess the stability of plateau values attained during irradiation. Experiments were conducted in triplicate for each of the investigated hydrogel formulation in a strain-controlled mode at constant temperature (25°C), shear strain (0.1), and frequency (1 Hz). The gel time was defined as the time of crossover of *G*′ and *G*″, which was determined as the instant at which *G*′ overcomes *G*″ (Lutolf and Hubbell, 2003).

#### Mechanical Properties

Samples were photo-crosslinked *in situ* between rheometer plates (same configuration as described in section “Gelation Kinetic”) and characterized in their relaxed state (i.e., immediately after photo-crosslinking) at room temperature. The following tests were performed:

1. shear strain amplitude sweep in the range of 0.001–0.15 shear strain (at a constant frequency of 1 Hz), to assess samples linear viscoelastic region (LVR);
2. frequency sweep in the range of 0.01–10 Hz (at a constant shear strain of 0.05), to obtain samples *G*′ and *G*″ frequency spectra;
3. creep recovery with creep and recovery phases, respectively, of 300 and 600 s (at a constant shear stress corresponding to 0.05 shear strain) to investigate samples creep behavior.

### Swelling Properties

Cylindrical (5-mm diameter, 3-mm height) hydrogel samples were obtained by irradiating 60 μL of precursor solutions (460 nm, 140 mW/cm^2^) for 600 s. Irradiation was performed in custom molds allowing easy sample collection with minimal stress after photo-crosslinking. Hydrogel relaxed weight (*W*_*g,r*_) was determined immediately after photo-crosslinking. Samples were then incubated in PBS 1× at 37°C and weighed every 12 h until a constant weight was reached, that is, the equilibrium swollen weight, *W*_*g,s*_. Finally, samples were freeze-dried (−50°C, 0.45 mbar) for 72 h to obtain their freeze-dried weight, *W*_*g,dry*_. The volume of the gel in a given state (*V*_*g,state*_, i.e., dry, relaxed or equilibrium swollen) was calculated using the following equation (Eq. 1):

(1) $$V_{g,s\,t\,a\,t\,e}=\frac{\left(W_{g,s\,t\,a\,t\,e}-W_{g,d\,r\,y}\right)}{\rho_{w}}+\frac{W_{g,d\,r\,y}}{\rho_{p}}$$

In the equation, *W*_*g,state*_ is the weight of the hydrogel in the given state, *ρ_w_* is the density of the swelling media (i.e., PBS 1×, approximated as that of water), and *ρ_p_* represents the density of the polymer in the freeze-dried state (taken as the mass averaged density of hydrogel solid components). The right addend in (1) represents the volume of the freeze-dried polymer (*V*_*p*_ = *V*_*g*,*dry*_), whereas the equilibrium volume swelling ratio is defined as *Q*_*eq*_ = *V*_*g*,*s*_/*V*_*g*,*dry*_.

### Analysis of Hydrogel Network Structure

The average molecular weight between crosslinks ($\bar{M_{c}}$) and the mesh size (ξ) of the hydrogels were calculated from either equilibrium swelling or mechanical data using variations of the Bray–Merrill model, as described in section “Analysis of Hydrogel Network Structure” in Supplementary Material.

### Amine Reaction Characterization

A second-step amino-crosslinking reaction was used to increase the elastic modulus of hydrogel, with incubation of hydrogels with LOx or with glyoxal (GlyO). Lysyl oxidase (LOx) solution was prepared with 0.1 U/mL of LOx and supplemented with 5 μg/mL of L-ascorbic acid and 5 μg/mL of CuSO_4_ as cofactors (Elbjeirami et al., 2003). GlyO, the smallest dialdehyde compound, was here used as a positive control (non-enzymatic Schiff base formation) and stoichiometrically administered to have a 1:1 aldehyde:amine molar ratio. To evaluate the feasibility of this approach, LOx and GlyO reactions were first tested with un-crosslinked 0.75 A/DA hydrogel precursor solutions (where both amino crosslinker and substrates have high mobility) by measuring the free amino content over time and after 0, 0.5, 1, 3, and 18 h of reaction. Amines content was measured using the ninhydrin assay (section “Amine Reaction Characterization in Soluble Models and Hydrogels” in Supplementary Material). A similar procedure was used with hydrogels after incubation with LOx and GlyO, measuring the free amino content over time (i.e., after 0, 1, 3, 18, and 48 h of reaction) with a modified ninhydrin assay protocol (section “Amine Reaction Characterization in Soluble Models and Hydrogels” in Supplementary Material) to confirm the diffusion of LOx and GlyO and consequent additional crosslinking of hydrogels.

### Unconfined Compression Tests

Cylindrical hydrogels (5-mm diameter, 3-mm height) were photo-crosslinked as described above. After polymerization, samples were placed in high-performance liquid chromatography glass vials and submerged in 140 μL of LOx or GlyO amino crosslinking solution. Samples were incubated for 48 h at 37°C to complete the reaction. Hydrogels submerged in PBS 1× were used as control. After incubation, samples were mechanically tested in their equilibrium swollen state. Uniaxial unconfined compression tests were performed at room temperature using a TA.XTplus Texture Analyser (Stable Microsystem, Mississauga, Canada). Compressive tests were performed at a constant strain rate of 0.01 s^–1^ with samples partially immersed in PBS 1× to preserve their hydration during measurements (Kane and Roeder, 2011; Mattei and Ahluwalia, 2016). Prior to testing, samples were carefully measured in thickness (*l*_*0*_) and diameter (*d*) with a caliper: dimensions were determined averaging at least three different measurements. Force (*F*) and displacement data (*l*) were recorded starting with a zero stress initial condition (Mattei et al., 2014b; Tirella et al., 2014). Force and actual compressive displacement data were respectively normalized to the cross-sectional area (π*d*^2^/4) and the initial length of the sample (*l*_*0*_), obtaining the engineering stress (σ) and strain (ε). Compressive moduli were derived as the slope of the first linear portion of the σ−ε curve (Mattei et al., 2015), here defined as the region in which the stress varies linearly with the applied strain giving a *R*^2^ ≥ 0.995.

### Cell Culture

The human hepatocellular carcinoma HepG2 cell line was purchased from ATCC (American Type Culture Collection) and cultured in Eagle minimal essential medium (EMEM; Sigma-Aldrich) supplemented with 10% (vol/vol) fetal bovine serum (Sigma-Aldrich), 1% (vol/vol) non-essential amino acids, 1% (vol/vol) EMEM vitamins, 2 mM L-glutamine, 100 U/mL penicillin, and 100 μg/mL streptomycin (Invitrogen, Monza, Italy). Cells were grown in standard conditions (37°C, 5% CO_2_) in 75-cm^2^ tissue culture flasks. At confluence, cells were washed with PBS 1×, detached with a solution of 0.05% trypsin and 0.02% EDTA (Sigma-Aldrich) in PBS 1× for 10 min, pelleted by centrifugation at 1,500 rpm for 5 min and finally resuspended with fresh medium to the desired cell density. Cells were cultured and used until passage 20 and then discarded.

Biocompatibility of functionalized 0.50 to 0.10 A/DA-NH_2_/A_PEGDA_ hydrogels was tested using HepG2 cells either seeded on the top of hydrogels or encapsulated within the same hydrogel type, hereafter referred as “2D model” and “3D model,” respectively. Perfusion experiments were performed using a variation of LiveBox1 bioreactor (IVTech s.r.l., Massarosa, Italy) connected in a closed-loop circuit with a peristaltic pump (Ismatec IPC-4; Ismatec SA, Zurich, Switzerland) and a mixing chamber via sterile silicone tubing following manufacturer’s instruction.

#### 2D *in vitro* Models

The 0.50 to 0.10 A/DA-NH_2_/A_PEGDA_ hydrogel precursor solution was prepared in PBS 1× (pH 7.4) and sterile filtered with a 0.22-μm filter. A volume of 250 μL of precursor solution was gently pipetted onto the LiveBox1 glass bottom, irradiated (460 nm, 140 mW/cm^2^, 10 min) forming hydrogels with a diameter of 15 mm and thickness of about 1.4 mm, washed with PBS 1×, and then seeded with 50,000 HepG2 cells suspended in 0.5 mL of complete medium. As control, a volume of 250 μL of 2.7 mg/mL collagen solution was pipetted onto the LiveBox1 glass bottom, incubated (37°C, 5% CO_2_) obtaining collagen hydrogels with diameter of 15 mm and thickness of about 1.4 mm, washed with PBS 1×, and then seeded with 50,000 HepG2 cells suspended in 0.5 mL of complete medium. LiveBox1 culture chambers were closed with PDMS top, clamped as per the manufacturer’s instructions, and then transferred in incubator for standard cell culture (37°C, 5% CO_2_). After 24 h, 2D models were perfused by connecting the LiveBox1 to a peristaltic pump.

#### 3D *in vitro* Models

Equal volumes of cell suspension and hydrogel precursor solution (both prepared at double of their final concentrations) were gently mixed to obtain homogeneous 0.50 to 0.10 A/DA-NH_2_/A_PEGDA_ precursor solution containing 5 × 10^6^ HepG2/mL. A volume of 250 μL of cell precursor suspension was gently pipetted in LiveBox1 culture chamber and photo-crosslinked (460 nm, 140 mW/cm^2^, 10 min), obtaining hepatocyte-laden hydrogels with 15-mm diameter and about 1.4-mm thickness. Hydrogels encapsulating HepG2 were washed with PBS 1×, and 0.5 mL of complete culture medium was added in the culture chamber. LiveBox1 culture chambers were closed with PDMS top, clamped as per the manufacturer’s instructions, and then transferred in incubator for standard cell culture (37°C, 5% CO_2_). After 24 h, 3D models were perfused by connecting the LiveBox1 to a peristaltic pump.

#### Cell Culture Protocols and Analyses

It is noteworthy to mention that in both 2D and 3D experiments the bioreactor circuit was filled with 7 mL of complete culture medium and perfused at a flow rate of 200 μL/min, resulting in a laminar flow on the hydrogels surface and guaranteeing a good trade-off between nutrient volumetric mass transport and shear stress on cultured cells (Mattei et al., 2014a, 2017; Giusti et al., 2017). Cell viability was measured at days 1, 3, and 7 using the CellTiter-Blue assay (2D models, section “CellTiter-Blue Viability Assay” in Supplementary Material) or live/dead assay (3D models, section “Live/Dead Fluorescence Viability Testing” in Supplementary Material). Cell morphology for 2D and 3D models was analyzed at day 7 with DAPI/phalloidin immunofluorescence staining (section “Immunofluorescence Staining” in Supplementary Material). Cell culture media were collected after 1, 3, and 7 days of culture for both 2D and 3D models and stored at −80°C for albumin and urea quantification (section “Assessment of Hepatocyte Metabolic Function” in Supplementary Material).

### Statistical Analysis

Hydrogel characterization experiments were carried out on a minimum of three independent preparations. Cell culture experiments were performed with biological duplicates (minimum of *n* = 4 samples). All results were reported as the mean ± standard deviation, unless otherwise noted. The statistical significance of differences between experiments was determined using a two-tailed Student *t-*test when comparing two groups of data or one-way analysis of variance (ANOVA) followed by Tukey *post hoc* multiple-comparisons test when comparing multiple groups. Differences were considered significant at *p* < 0.05. Statistical analysis was performed using OriginPro (OriginLab, Northampton, MA, United States).

## Results and Discussion

### Rheology and Gel Formation

**FIGURE 1:**
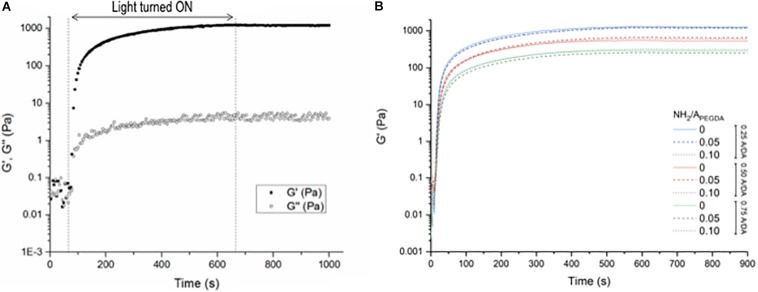
Shear rheology measurements during hydrogel photo-crosslinking. Samples were irradiated for 600 s (460 nm, 140 mW/cm^2^); measurements were performed up to 900 s. **(A)** Storage (*G*′) and loss (*G*″) shear moduli measured for the 0.50–0.10 A/DA-NH_2_/A_PEGDA_ hydrogel formulation as a function of time. The irradiation period is displayed between the dashed lines. Samples were monitored for additional 300 s after irradiation. **(B)** Variation of *G*′ during and after light exposure for all investigated hydrogel formulations: 0.25 A/DA ratio (blue), 0.50 A/DA ratio (red) and 0.75 A/DA ratio (green); no hydrogel amino content (solid line), hydrogel amino contend NH_2_/A_PEGDA_ molar ratio = 0.05 (dashed line) and hydrogel amino contend NH_2_/A_PEGDA_ molar ratio = 0.10 (dotted line). Each curve is the average of measurements performed on *n* = 3 independent samples per formulation. Time 0 is here defined as the instant at which the light was switched on. Statistical analysis: one-way ANOVA considering plateau values of *G*′, *p* < 0.05.

Storage (*G*′) and loss (*G*″) shear moduli were recorded over time during irradiation, monitoring the gelation kinetics *in situ*: a typical experimental curve is shown in Figure 1A. The gel point, evaluated as the time at which *G*′ overcomes *G*″, was reached within 20 s of light exposure for all the investigated hydrogels. Once the gel point was reached, both shear moduli increased until reaching plateau values (approximately after 400-s irradiation), which remain stable over time even after irradiation, indicating the completion of hydrogel crosslinking during the photopolymerization process. The time evolution of *G*′ for all investigated hydrogel formulations is reported in Figure 1B. Mechanical properties were found independent of the hydrogel amino content (expressed as NH_2_/A_PEGDA_ molar ratio) and dictated only by the A/DA ratio. These results meet the initial design criteria and confirm the required characteristics of hydrogel stiffness proportional to the density of diacrylates/crosslinks.

### Shear Mechanical Characterization

**FIGURE 2:**
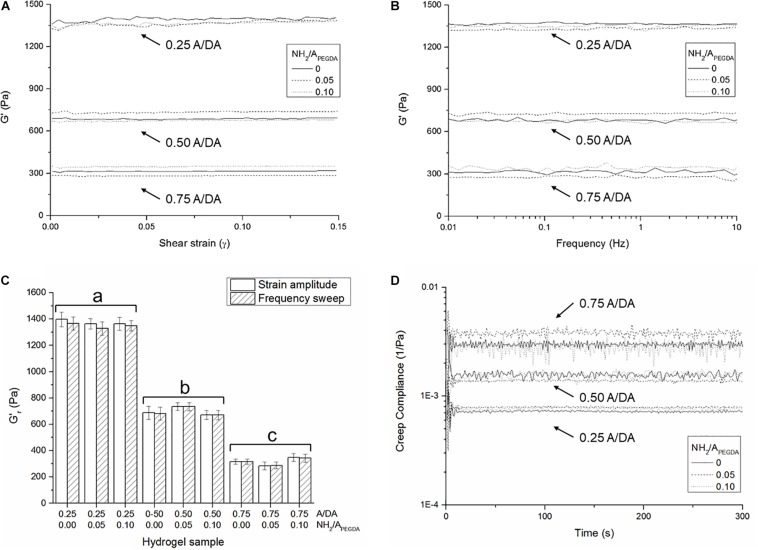
Averaged storage shear moduli (*n* = 3 independent samples per formulation) measured as a function of **(A)** shear strain and **(B)** applied frequency, for all investigated hydrogel formulations. **(C)** Hydrogel shear storage moduli in the relaxed state ($G_{r}^{{}^{\prime }}$) derived from both strain amplitude and frequency sweep measurements. Different letters indicate significant differences between samples (one-way ANOVA, *p* < 0.05). **(D)** Shear creep compliance during the creep phase. Note that the initial oscillations at the beginning of the creep experiments are likely due to the inertia of the measuring instrument (Baravian and Quemada, 1998).

Typical shear strain amplitude and frequency sweep experimental results are shown in section “Shear Strain Amplitude and Frequency Sweep” in Supplementary Material. No significant changes in *G*′ were observed for any of the investigated samples either with shear strain (Figure 2A) or frequency (Figure 2B), indicating that the LVR for all hydrogels extended at least up to 0.15 shear strain and confirming their substantially elastic behavior (*G*″ values are also frequency-independent and <<*G*′, data not shown). These strain- and frequency-independent values of *G*′ were then used to derive hydrogel shear moduli in the relaxed state ($G_{r}^{\prime }$). No statistically significant differences were observed between values derived from either strain amplitude or frequency sweep measurements (Figure 2C). Shear storage moduli were dictated only by the A/DA molar ratio, regardless of the amount of tethered amines. This observation was in line with our findings on gelation kinetics and expected as in agreement with initial hypothesis. Measured $G_{r}^{\prime }$ values were found to be statistically different between hydrogel groups (*n* = 3, one-way ANOVA, *p* < 0.05) for 0.25, 0.50, and 0.75 A/DA hydrogel families with values of 1.36 ± 0.05, 0.70 ± 0.04, and 0.32 ± 0.03 kPa, respectively.

Creep-recovery results further confirmed the substantially elastic behavior observed (typical experimental creep-recovery angle over time plotted in section “Creep-Recovery” in Supplementary Material). The shear creep compliance (*J*) for all investigated hydrogels was found constant over time during the creep phase (Figure 2D) and correlated well with the respective values of $G_{r}^{\prime }$ derived from either strain amplitude or frequency sweep tests (Figure 2C), being $J=1/G_{r}^{\prime }$. In particular, shear storage moduli in the relaxed state were estimated from creep compliance and were found to be statistically different between hydrogel groups (*n* = 3, one-way ANOVA, *p* < 0.05). Values for 0.25, 0.50, and 0.75 A/DA hydrogel families were 1.31 ± 0.06, 0.66 ± 0.05, and 0.31 ± 0.05 kPa, respectively.

### Swelling and Hydrogel Network

**FIGURE 3:**
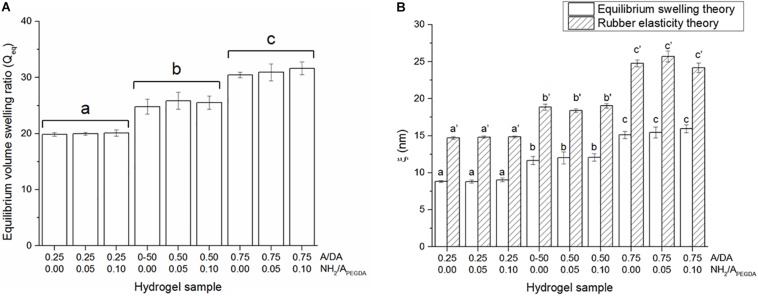
**(A)** Equilibrium volume swelling ratios (*Q*_*eq*_). Different letters indicate significant differences between samples (one-way ANOVA, *p* < 0.05). **(B)** Hydrogel mesh size estimations (ξ) obtained either with equilibrium swelling and rubber elasticity theories. Different letters indicate significant differences between samples (one-way ANOVA, *p* < 0.05). Letters with no sign are referred to ANOVA results of ξ calculated using equilibrium swelling data, whereas those with an apostrophe indicate ANOVA results obtained for ξ estimated from mechanical data.

Equilibrium volume swelling ratio (*Q*_*eq*_) was also found to be dependent only on the hydrogel A/DA molar ratio, regardless of the amount of tethered amines (Figure 3A), in agreement with both rheological results and initial hypothesis. Equilibrium volume swelling ratios were found to be statistically different between hydrogel groups and with values for 0.25, 0.50, and 0.75 A/DA hydrogel groups of 20.0 ± 0.3, 25.4 ± 1.1, and 31.0 ± 1.0 respectively. Hydrogel mesh size (ξ) estimations are shown in Figure 3B. Although ξ values derived from equilibrium swelling were significantly lower than those obtained from rubber elasticity theory for all samples investigated (two-tailed Student *t*-test, *p* < 0.05), both series exhibited the same trend, consistent with data reported in other studies (Lin and Freeman, 2006; Beamish et al., 2010). A correlation between hydrogel mesh size and A/DA molar ratio was found and, as expected, independent of the amount of tethered amines (i.e., NH_2_/A_PEGDA_). These estimations were also in agreement with mechanical and swelling data. It is worth noting that the models used for ξ estimations work well for simple hydrogels and uniform networks (Lutolf and Hubbell, 2003; Zustiak and Leach, 2010), whereas assumptions could be required while calculating ξ in the case of more complex hydrogel networks, such as those investigated in this work. Therefore, caution should be exerted in overinterpreting the results obtained.

### Enzymatic (LOx) and Non-enzymatic (GlyO) Reactions

**FIGURE 4:**
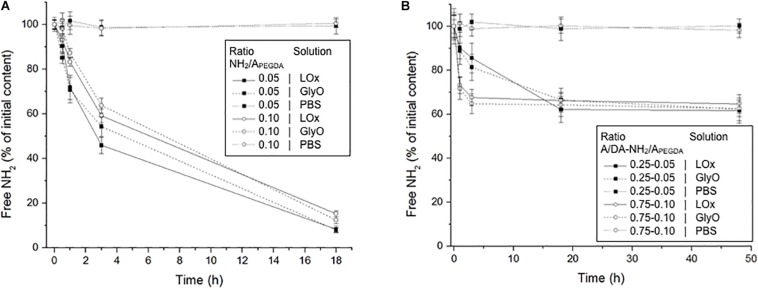
**(A)** Free amino content measured over time for both the 0.75 to 0.05 (black squares) and the 0.75 to 0.10 (white circles) A/DA-NH_2_/A_PEGDA_ soluble models incubated with LOx, GlyO (positive control) and PBS (negative control). **(B)** Free amino content measured over time for both the 0.25 to 0.05 (black squares) and the 0.75 to 0.10 (white circles) A/DA-NH_2_/A_PEGDA_ crosslinked hydrogels incubated with LOx, GlyO or PBS. Each free amine time series was analyzed independently and found statistically different (one-way ANOVA, *p* < 0.05).

The incubation with either LOx or GlyO to crosslink available primary amines resulted in a significant decrease of free amino groups over time for both 0.75 to 0.05 and the 0.75 to 0.10 A/DA-NH_2_/A_PEGDA_ hydrogel precursor solutions, that is, soluble un-crosslinked models. GlyO was used as a non-enzymatic positive control. Both LOx- and GlyO-mediated reaction kinetics were similar for the tested soluble models, with about 85% of initial free NH_2_ reacted after 18 h (Figure 4A), thereby indicating that both LOx and GlyO were capable of reacting with free NH_2_ provided by CysAm-PEG-acrylate macromers. No significant variations in NH_2_ content over time were observed for negative controls in absence of amino crosslinker. Notably, both LOx and GlyO, on the one hand, and the amine-containing substrates, on the other, have high mobility in these liquid (soluble) models, with no significant limitations in diffusion and in spatial constraints/hindrance. This is likely not the case when considering crosslinked hydrogel networks, but nevertheless a significant decrease of free amines over time was also observed when photopolymerized materials were exposed to LOx or GlyO (one-way ANOVA, *p* < 0.05; each free NH_2_ time series was analyzed independently). Faster kinetics was observed in 0.75 to 0.10 A/DA-NH_2_/A_PEGDA_ hydrogels than in 0.25 to 0.05 ones (Figure 4B), as expected. No differences were observed between LOx and GlyO amine reaction kinetics, in agreement with results obtained for soluble models. The final amino conversion was found to be independent of the hydrogel formulation and the crosslinking reaction (i.e., LOx, GlyO), with about 40% of initial free NH_2_ reacted after 48 h. As expected, the reaction efficacy in hydrogels was worse than in soluble models in terms of both kinetics and amino conversion at plateau (slower, reduced number of crosslinks formed). We believe that this difference could be due to (i) diffusional limitations of both crosslinking agents within crosslinked hydrogel networks and (ii) low mobility of free amines in photo-crosslinked hydrogels, which are covalently linked to the hydrogel network and hence constrained and/or partially hindered in a 3D environment. Faster reaction kinetics were observed for 0.75 to 0.10 A/DA-NH_2_/A_PEGDA_ gels with respect to 0.25 to 0.05 ones, possibly because of the larger mesh size and a higher amino content of the former hydrogels. From the similar amino reaction kinetics and plateau conversion observed between LOx and GlyO, it is possible to conclude that the enzyme, although significantly larger than GlyO, can effectively diffuse within hydrogels and react with free amino groups similarly to the positive control. Assuming a globular protein model, the LOx used in this work (MW 116 kDa) was estimated to have a diameter of 9 nm with the Malvern ZetaSizer software (Malvern Instruments Ltd., Malvern, United Kingdom), thus suggesting that enzyme-responsive hydrogel mesh sizes (ξ) should be sufficiently large to allow the enzyme diffusion, consistently with *ξ* estimations.

Because LOx reaction kinetics and amine conversion at plateau were found to be surprisingly similar to those observed administering GlyO in a stoichiometric aldehyde:amine molar ratio, further studies were performed administering GlyO at 5:1 and 10:1 aldehyde:amine molar ratios to 0.25 to 0.05 A/DA-NH_2_/A_PEGDA_ hydrogels: increasing GlyO concentration resulted in faster reaction kinetics, as expected because the amine-glyoxal reaction is of order 2 (section “Glyoxal Amino Crosslinking Reaction in 0.25–0.05 A/DA-NH2/APEGDA Gels at 5:1 and 10:1 Aldehyde:Amine Molar Ratios” in Supplementary Material). These results confirmed our initial hypothesis to use LOx to direct the on-demand gelation and further stiffen hydrogels, as LOx is an enzyme typically involved in ECM pathological crosslinking/stiffening (Desmoulière et al., 1997; Perepelyuk et al., 2013) and would better mimic the hepatic healthy-to-fibrotic ECM-stiffening process observed *in vivo*.

### On-Demand Hydrogel Stiffening

Engineered enzyme-responsive PEG-based hydrogels were designed to stiffen progressively and with a dual strep gelation: the first step defining the initial mechanical properties (i.e., photopolymerization) and the second on-demand step engineered to modifying/tuning the resultant hydrogel stiffness (LOx, GlyO). Decreased shear modulus (*G*′) and increased equilibrium swelling ratio (*Q*_*eq*_) were observed when the ratio between acrylate groups belonging to the monoacrylate PEG derivatives and to the diacrylate PEG-derivatives (i.e., A/DA) was increased in the hydrogel precursor solution. In all the studies, the total PEG concentration was kept constant to 5% wt/vol. Moreover, increased hydrogel mesh size (ξ) was found proportional to increase in A/DA ratio, in agreement with published reports on PEGDA-co-PEGMA copolymers (Lin and Freeman, 2006; Beamish et al., 2010). This could be due to the presence of mono-acrylate PEG derivatives that do not form crosslinks, but act as pendant chains within the hydrogel network and thus may decrease the crosslinking density (Beamish et al., 2010).

**FIGURE 5:**
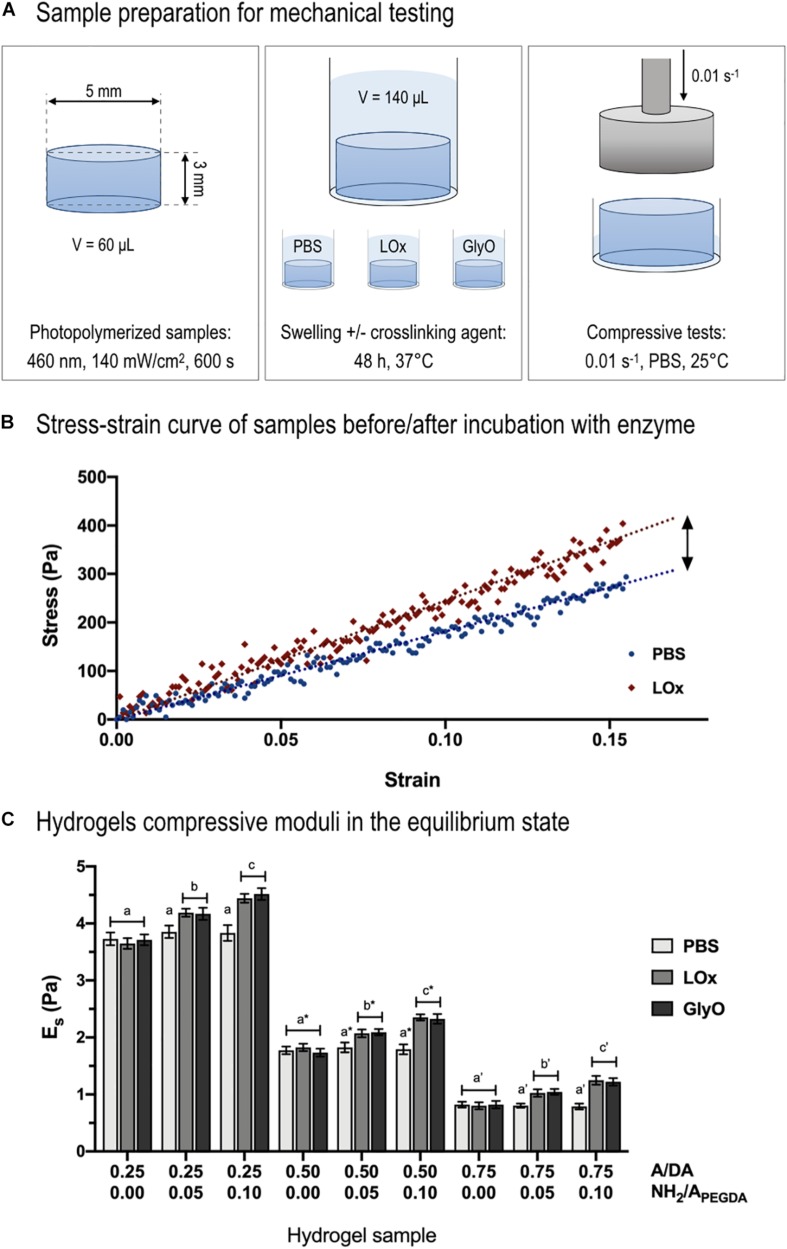
Compressive mechanical tests. **(A)** Schematic representing sample preparation for mechanical testing. **(B)** Example of stress–strain curve of 0.50 to 0.10 A/DA-NH_2_/A_PEGDA_ hydrogel before (blue dots) and after incubation with LOx (red diamonds). Typical linear fitting curve data within 15% strain (blue and red dotted lines) are displayed to show the linear (elastic) behavior of hydrogels during compression. **(C)** Hydrogel compressive moduli (*E*_*s*_) measured after incubation with LOx, GlyO (positive control) or PBS (negative control). One-way ANOVA was performed considering each A/DA hydrogel family independently, with differences *p* < 0.05 considered statistically different. Letters with no sign indicate ANOVA results obtained analyzing the 0.25 A/DA hydrogel family, whereas letters with an asterisk and those with an apostrophe are referred to ANOVA results obtained for the 0.50 and the 0.75 A/DA families, respectively. Different letters indicate significant differences between samples (*p* < 0.05).

Mechanical tests in unconfined compression were performed on sample incubated for 48 h in LOx, GlyO (positive control), and PBS 1× (negative control) to investigate and validate whether LOx and GO amino reactions were able to create new covalent crosslinks (Figure 5A). Samples were tested at low strain rates (0.01 s^–1^) as it was previously shown that an increase in compressive strain rate results in an increase of apparent stiffness when using soft and hydrated samples (Mattei et al., 2014b; Tirella et al., 2014). Hydrogel bulk compressive moduli in the equilibrium swollen state (*E*_*s*_) were measured calculating the slope of stress–strain curves in their first linear region (0.05 strain, Figure 5B). Of note, the linear region extended up to 0.15 strain for all samples. A significant increase in *E*_*s*_ was observed for amine-containing hydrogels after 48-h incubation in either LOx or GlyO (positive control) solutions with respect to PBS (negative control) and found proportional to the number of tethered amines (Figure 5C). As expected, no variation in compressive moduli was observed in amine-free hydrogels (i.e., with NH_2_/A_PEGDA_ = 0.00) incubated with the crosslinkers and PBS.

Shear moduli in the equilibrium swollen state (i.e., $G_{s}≅G_{s}^{\prime }$) for control samples incubated 48 h in PBS were derived from compressive stress–strain data, finding non-significant differences with respect to those estimated from shear measurements in the relaxed state after photo-crosslinking (section “Calculated Equilibrium Swollen Hydrogel Shear Moduli from Compressive Data” in Supplementary Material). Finally, the Poisson ratio, calculated as ν = (*E*_*s*_/2*G*_*s*_)−1, was found to be 0.51 ± 0.07 for the investigated hydrogels, in agreement with the theoretical value of 0.5, generally assumed for PEG-based hydrogels (Elbert and Hubbell, 2001; Liao et al., 2008).

The normalized hydrogel stiffening (Δ*E*_*s*_/Δ*NH*_2_) was found to be independent of both hydrogel type and amino crosslinker and equal to 126 ± 13 Pa per mmol of reacted amines. This result suggested that the ratio between crosslinking forming amines (unknown) and total reacted amines (experimentally measured) should be similar for all investigated amino-containing hydrogels and independent of the crosslinker type. Samples incubated in PBS showed no differences in stiffness with respect to that after photo-crosslinking, confirming negligible sample degradation (West and Hubbell, 1999). Compressive mechanical and rheological moduli confirmed that hydrogels properties could be decoupled from the amount of tethered primary amines, as hypothesized. Therefore, by varying the A/DA molar ratio in the hydrogel precursor solution, it was possible to tune the initial hydrogel mechanical properties regardless of the amount of tethered substrate, essential instead for the subsequent enzymatic stiffening reaction.

### Cell Viability, Morphology, and Functional Properties

The rapid hydrogel gelation (<20 s) was found favorable for 3D cell encapsulation, allowing homogeneous cell distribution throughout the hydrogel volume and avoiding cell settling, as previously reported for longer gelation kinetics. HepG2 viability and function were not significantly affected by the first photopolymerization step (i.e., 400–500 nm, 140 mW/cm^2^, 10 min), in line with what that previously reported on the effects of UV irradiation (Skardal et al., 2010) and visible light (Bahney et al., 2011; Turturro et al., 2013).

**FIGURE 6:**
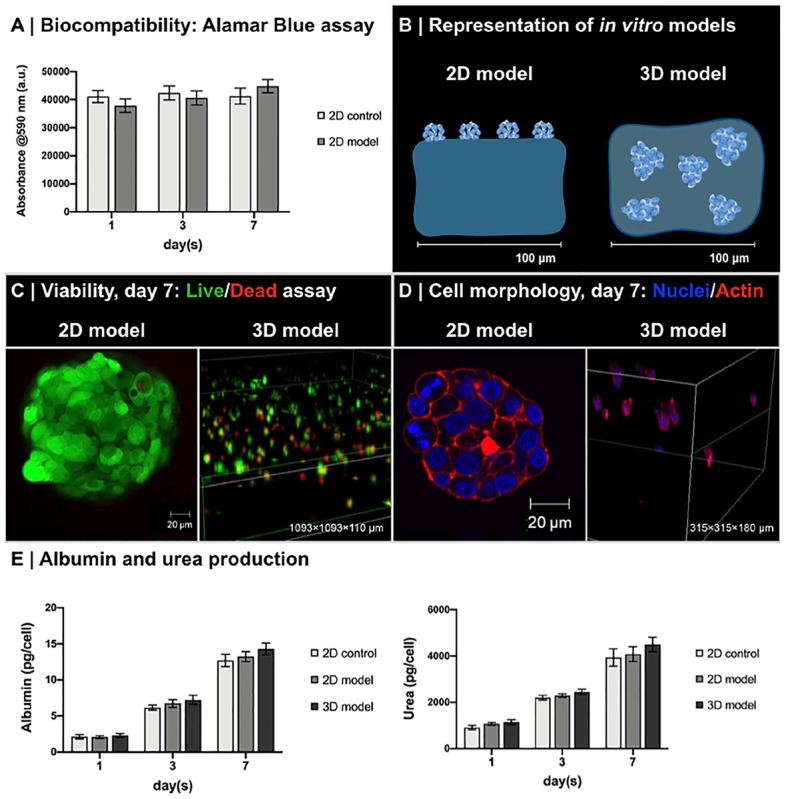
Biocompatibility of enzyme-responsive material. **(A)** Viability of HepG2 seeded on the top of 0.50 to 0.10 A/DA-NH_2_/A_PEGDA_ (2D model) and collagen gels (2D control). Data are reported as average ± standard deviation (*n* = 4 independent experiments). No significant differences were observed at different time points (one-way ANOVA, *p* < 0.05). **(B)** Representation of *in vitro* 2D model and 3D model with HepG2. **(C)** Live/dead assay of HepG2 in 2D and 3D models. Clusters of live (green) and dead (red) HepG2 cells were observed in both 2D models (detail of HepG2 on 0.50 to 0.10 A/DA-NH_2_/A_PEGDA_) and in 3D models (volumetric rendering of HepG2 in 0.50 to 0.10 A/DA-NH_2_/A_PEGDA_ hydrogels, large view). **(D)** HepG2 morphology in 2D and 3D models. Immunofluorescence staining for cell nuclei (DAPI, blue) and F-actin filaments (phalloidin, red): clusters of HepG2 were observed in both 2D models (detail of HepG2 on 0.50 to 0.10 A/DA-NH_2_/A_PEGDA_) and in 3D models (volumetric rendering of HepG2 in 0.50 to 0.10 A/DA-NH_2_/A_PEGDA_ hydrogels, large view). **(E)** Albumin and urea production normalized against the number of HepG2 over time for 2D model and 3D model. Collagen gels with HepG2 seeded on the top were used as a control (2D control). Data are reported as average ± standard deviation (*n* = 4 independent experiments). No significant differences were observed at different time points (one-way ANOVA, *p* < 0.05). Of note, albumin was produced with an average rate of 1.99 ± 0.16 pg/cell per day (2D control), 2.07 ± 0.18 pg/cell per day (2D model), and 2.25 ± 0.19 pg/cell per day (3D model), whereas urea was produced with an average rate of 736.57 ± 174.56 pg/cell per day (2D control), 806.68 ± 246.89 pg/cell per day (2D model), and 866.70 ± 253.41 pg/cell per day (3D model). Production rates of both albumin and urea were similar in the models tested with respect to control.

Biocompatibility studies were performed on 0.50 to 0.10 A/DA-NH_2_/A_PEGDA_ hydrogels with HepG2. This hydrogel was selected because of the initial stiffness similar to that of decellularized liver matrix (Mattei et al., 2014b). No differences were observed between 0.50 and 0.10 A/DA-NH_2_/A_PEGDA_ and collagen hydrogels at any time point and up to 7 days (Figure 6A). Live/Dead confocal acquisitions after 7 days of culture on 2D models confirmed a viability greater than 95% of cells, with a typical reorganization of HepG2 into cell clusters (Figures 6B,C), known behavior of HepG2 cells (Vogel, 2006). After 7 days of culture, also 3D models showed high viability (Live/Dead, day 7; Figure 6C), as well as homogeneous distribution/dispersion throughout the hydrogel (Figures 6C,D). Cell viability within 3D models was found slightly lower than 2D models and approximately 60% with respect to the control; again this was already reported in literature for encapsulated cells in hydrogels (Rios De La Rosa et al., 2018; Almari et al., 2019) and for HepG2 (5 × 10^6^ cell/mL) within physically crosslinked agarose gels (Park et al., 2010). HepG2 were homogeneously distributed throughout the hydrogel volume and exhibited a spherical configuration with regions of cluster formation, consistent with other reports (Ise et al., 1999; Jones et al., 2009). Notably, we observed a decrease in the number of viable cells with increasing the distance from the surface of the hydrogel in contact with the culture medium. This observation was in accordance with published reports and expected due to nutrient transport limitations (Cuchiara et al., 2010; Giusti et al., 2017; Mattei et al., 2017).

Hepatocyte (HepG2) functionality was also tested as simple production of albumin and urea at different time points (days 1, 3, and 7). No significant differences were found between 2D and 3D models at tested time points (Figure 6E). Values were normalized against cell number, considering negligible HepG2 proliferation after 1 week of culture (Lan et al., 2010; Corstorphine and Sefton, 2011), with 50,000 cells for 2D models (initial seeding number) and 750,000 cells for 3D models (60% of initially encapsulated HepG2), reporting data consistent with other reports (Guzzardi et al., 2009; Vinci et al., 2010; Mueller et al., 2011; Rossouw et al., 2012). Moreover, these results suggest that both urea [Stokes radius, *r*_*s*_ = 0.28 nm (Hirche et al., 1975)] and albumin [*r*_*s*_ = 3.58 nm (Roheim et al., 1979)] produced by HepG2 cells can effectively diffuse through the hydrogel network, in agreement with our mesh size estimations and the results reported by Underhill et al. (2007) for similar gels. Results suggested that engineered enzyme-responsive PEG-based hydrogels are biocompatible and sustain HepG2 functionality and could be potentially used as *bioinks* for 3D *in vitro* models and biomedical applications.

## Conclusion

We report on an engineered PEG-based enzyme-responsive hydrogel precursors to model soft tissue and its stiffening for the development of 3D hepatic *in vitro* models. The hydrogels developed in this study sustain hepatocyte viability and maintain cell function both in 2D and 3D *in vitro* models; coupled with the possibility of a responsive hardness control, this makes these biomaterials promising to further study the effect of stiffening in early stage models of fibrotic pathologies. The approach herein presented is easily adapted to tailor initial mechanical properties of other soft tissues and in the range of 1–20 kPa, as well as to engineer the stiffening step to mimic specific pathophysiological mechanical changes. These biomaterials can be used as *bioinks* for the design of 3D *in vitro* models, with advantages in understanding tissue pathologies and disease mechanisms and potentially test and predict drug efficacy/toxicity to validate new therapies.

## Data Availability Statement

The raw data supporting the conclusions of this article will be made available by the authors, without undue reservation, to any qualified researcher.

## Author Contributions

AT, GM, and NT contributed conception and design of the study. AT, GM, and ML contributed equally on acquisition, analysis or interpretation of data for the work. GM organized the database. GM and AA performed the statistical analysis. AT and GM wrote the first draft of the manuscript. ML wrote sections of the manuscript. All authors contributed to manuscript revision, read and approved the submitted version.

## Conflict of Interest

GM and AA are co-founders and shareholders of IVTech s.r.l. They are not employed at IVTech s.r.l. nor received any salary, stock, or bonus from the company either to perform or during the study. The company did not have any role in the study design, data collection and analysis, decision to publish, or preparation of the manuscript. The remaining authors declare that the research was conducted in the absence of any commercial or financial relationships that could be construed as a potential conflict of interest.
