# Supplementary material for: Functionalized Enzyme-Responsive Biomaterials to Model Tissue Stiffening in vitro
Source: Front Bioeng Biotechnol. 2020 Apr 8;8:208. doi: 10.3389/fbioe.2020.00208 (PMC7156543; doi:10.3389/fbioe.2020.00208)
Supplement: Supplementary file 1 [file Data_Sheet_1.docx]

Supplementary Material

# Preparation of hydrogel precursor solutions

Hydrogel precursor solutions were prepared by linking integrin-binding peptide (RGD) or cysteamine (Cys) to poly(ethylene glycol) diacrylate (PEGDA) acrylates via Michael-type addition. After, poly(ethylene glycol) methyl ether acrylate (PEGMA) was mixed with functionalized PEGs to obtain the desired acrylate/diacrylate (A/DA) molar ratio. Finally, visible light photo-initiating compounds (i.e. Eosin Y, TEA, NVP) were included and the pH adjusted to 7.4 (Supplementary Figure 1).

**Supplementary Figure 1.** Schematic showing the two-step reaction used for the preparation of hydrogel precursor solutions.

The Michael-type addition yielded three classes of products: 1) *un-reacted PEGDA macromers (black)*, with free acrylates on both ends, hence capable of reacting during hydrogel photo-crosslinking; 2) *mono-functionalized PEG-acrylate macromers (blue)*, with one end functionalized by a thiol compound (i.e. RGD-containing peptide or cysteamine) and a free acrylate group on the other, through which they can be covalently incorporated within the hydrogel network during photo-crosslinking; and 3) *di-functionalized PEG macromers (red)*, with both ends functionalized with RGD-containing peptide or cysteamine. This class is an undesired product as it is unable to react link to the hydrogel network during photo-crosslinking.

To evaluate the amount of PEGMA to add for obtaining hydrogel precursor solutions with desired A/DA molar ratio, it is important to estimate the expected PEGDA products after the Michael-type additions. The molar amount of acrylates belonging to mono-acrylate PEG derivatives in the precursor solution (A) is given by the sum of moles of mono-functionalized PEG-acrylate macromers plus those of the PEGMA to add, while the moles of acrylates belonging to di-acrylate PEG-derivatives (DA) are given by 2 × mol of unreacted PEGDA. Because of the high ratio of acrylates to thiols, only a very small number of the functionalized PEGs will be unreactive due to di-functionalization (as also reported by Park et al. (Park, Tirelli, and Hubbell 2003)). The probability of PEG bi-functionalization can be estimated as the product of two mono-functionalization events. Considering the *worst-case* scenario of Michael-type addition between cysteamine and PEGDA with the higher thiol/acrylate molar ratio (i.e. SH/A_PEGDA_ = 0.10), the probability of PEG bi-functionalization is given by 0.10 × 0.10 = 0.01 (thiols introduced by the peptide were not considered here, since they are significantly lower than to those of cysteamine). Therefore, PEG bi-functionalization was neglected in this study and we assumed that almost all peptide and cysteamine in the precursor solution were incorporated as dangling ends of mono-functionalized PEG-acrylate macromers. The molar amount of PEGDA, PEGMA and cysteamine to prepare a volume $V$ (in mL) of hydrogel precursor solution can be calculated using the following equations (Supplementary Equations 1-3), with total PEG and RGD-containing peptide concentrations fixed to 5 % w/v and 250 μM, respectively.

| $PEGDA\cdot\mathrm{MW}_{PEGDA}+PEGMA\cdot\mathrm{MW}_{PEGMA}=0.05\cdot V$ | Supplementary Equation 1 |
| --- | --- |
| $\frac{A}{DA}=\frac{PEGMA+CysAmPEGA+PeptidePEGA}{2\cdot\left( PEGDA-CysAmPEGA-PeptidePEGA \right)}=x$ | Supplementary Equation 2 |
| $\frac{{NH}_{2}}{A_{PEGDA}}=\frac{CysAm}{2\cdot PEGDA}=y$ | Supplementary Equation 3 |

In these equations $MW$ stands for macromer molecular weight (in g/mol), while $PEGDA$, $PEGMA$ and $CysAm$ respectively represent the molar amounts of PEGDA, PEGMA and cysteamine necessary to prepare a volume $V$ of hydrogel precursor solution. Assuming a 100 % thiol conversion in the Michael-type addition. This is in agreement with Nair et al. (Nair et al. 2012) and confirmed by the Ellman’s assays; moles of cysteamine-PEG-acrylate ($CysAmPEGA$) and peptide-PEG-acrylate ($PeptidePEGA$) in Supplementary Equation 2 were considered equal to $CysAm$ (to be calculated) and $Peptide$ (i.e. $0.25 \left[ \frac{\mu mol}{mL} \right] \cdot V\left[ mL \right]$).

Therefore, the A/DA ratio was derived using the Supplementary Equation 4:

| $\frac{A}{DA}=\frac{PEGMA+CysAM+Peptide}{2\cdot\left( PEGDA-CysAm-Peptide \right)}=x$ | Supplementary Equation 4 |
| --- | --- |

Parametric solutions for $PEGDA$, $PEGMA$ and $CysAm$ are reported in Supplementary Equations 5-7:

| $PEGDA=\frac{0.05\cdot V+(1+2x)\cdot Peptide{\cdot\mathrm{MW}}_{PEGMA}}{\mathrm{MW}_{PEGDA}+\left( 2x-4xy-2y \right)\cdot\mathrm{MW}_{PEGMA}}$ | Supplementary Equation 5 |
| --- | --- |
| $PEGMA=\frac{0.05\cdot V-{\mathrm{PEGDA}\cdot\mathrm{MW}}_{PEGDA}}{\mathrm{MW}_{PEGMA}}$ | Supplementary Equation 6 |
| $CysAm=2y\cdot PEGDA$ | Supplementary Equation 7 |

Hydrogel precursor solutions were prepared by dissolving all components in 10 mM phosphate buffered saline (PBS 1×). To link either the RGD-containing peptide or the desired amount of cysteamine to the PEGDA, the reaction mixture was incubated for 1 h in the dark at room temperature, allowing the Michael-type addition to occur (Rydholm et al. 2008; Jabbari 2011; Park, Tirelli, and Hubbell 2003). Thiol-acrylate reaction was monitored with Ellman’s assay (Ellman et al. 1961) by measuring free thiol content over time every 15 minutes up to 3 hours. No free thiols were observed in the reaction mixture after 30 minutes, while no significant changes in thiol concentration were measured in absence of PEGDA acrylates for up to one hour in 10 mM PBS at pH 7.4 (one-way ANOVA, *p* > 0.05; data not shown). Therefore, no significant disulfide bond formation occurred within the first hour in absence of acrylates (likely due to the pKa value of the sulphydryl group of cysteamine and cysteine, respectively equal to 8.6 and 8.33 (Mezyk 1995)). Consequently, the complete disappearance of thiols observed in the reaction mixture was owed to Michael-type addition of thiol-compounds onto PEGDA acrylates, confirming 100 % thiol conversion, as expected, and further justifying the assumptions made in the calculations. After the Michael-type additions, the partially peptide- or cysteamine-functionalized PEGDA was not isolated from the reaction mixture. The latter was added with the calculated amount of PEGMA (to obtain the desired A/DA molar ratio) and the photo-initiating system compounds (i.e. the visible light sensitizer, Eosin Y; the initiator, TEA; the accelerator and co-monomer, NVP) according to Table 1 in the main manuscript, obtaining the hydrogel precursor solutions.

# Analysis of hydrogel network structure

## Equilibrium swelling theory

The average molecular weight between crosslinks ($M_{c}$) was calculated using the Flory-Rehner equation as modified by Peppas and Merril for hydrogels prepared in the presence of water (Supplementary Equation 8) (Peppas et al. 2000).

| $\frac{1}{M_{c}}=\frac{2}{M_{n}}-\frac{\left( \bar{v}/V_{1} \right)\left[ \ln\left( 1-v_{2,s} \right)+v_{2,s}+\chi_{1}v_{2,s}^{2} \right]}{v_{2,r}\left[ \left( v_{2,s}/v_{2,r} \right)^{1/3}-\left( 2/\varphi\right)\left( v_{2,s}/v_{2,r} \right) \right]}$ | Supplementary Equation 8 |
| --- | --- |

In this equation $M_{n}$ is the number average molecular weight of the un-crosslinked polymer (i.e. the molecular weight of the macromer), $\bar{v}$ is the specific volume of the polymer (equal to the reciprocal of the dry polymer density, $1/\rho_{p}$) and $V_{1}$ is the molar volume of the solvent (18 cm^3^/mol for water). The parameters $v_{2,r}$ and $v_{2,s}$ are the polymer volume fractions in the relaxed and swollen hydrogel state, respectively, and can be derived from swelling experiments as $v_{2,r}=V_{p}/V_{g,r}$ and $v_{2,s}=V_{p}/V_{g,s}$, where $V_{p}$ is the bulk polymer volume, while $V_{g,r}$ and $V_{g,s}$ represent the hydrogel volume in the relaxed (i.e. immediately after crosslinking) and equilibrium swollen state, respectively. The Flory-Huggins polymer-solvent interaction parameter ($\chi_{1}$) was assumed constant for all the hydrogel formulations investigated, neglecting any network defects in agreement with (Mellott, Searcy, and Pishko 2001; Avens et al. 2011; Lin et al. 2005; Beamish et al. 2010), and fixed to a value of 0.426 (Leach et al. 2003). The junction functionality ($\varphi$) for mesh size calculations was considered equal to 4 according to Lin et al. (Lin et al. 2005) and Beamish et al. (Beamish et al. 2010) and Avens et al. (Avens et al. 2011). Moreover, the term 2/$M_{n}$, representing the correction factor for chain ends, was neglected in Eq. S8, in agreement with several studies (Ma and Elisseeff 2005; Lin et al. 2005; Avens et al. 2011) suggesting that the correction factor for chain ends can be neglected in case of highly swollen networks with $Q_{eq}$ > 10.

The mesh size was calculated by first computing the root-mean-square end-to-end distance of the polymer chain in the unperturbed (solvent-free) state, as described by Canal and Peppas (Supplementary Equation 9) (Canal and Peppas 1989):

| $\left( \bar{r}_{0}^{2} \right)^{1/2}=l\left( 2M_{c}/M_{r} \right)^{1/2}C_{n}^{1/2}$ | Supplementary Equation 9 |
| --- | --- |

in which $l$ is the average bond length (1.46 Å for PEG (Mellott, Searcy, and Pishko 2001), i.e. the average between C-C and C-O bond lengths in its repeat unit [-O-CH_2_-CH_2_]), $M_{r}$ is the molecular weight of the repeat unit (44 g/mol for PEG) and $C_{n}$ is the characteristic ratio of the polymer (typically 4.0 for PEG (Mellott, Searcy, and Pishko 2001)). The hydrogel average mesh size was then calculated using Supplementary Equation 10.

| $\xi={v_{2,s}^{-1/3}\left( \bar{r}_{0}^{2} \right)}^{1/2}$ | Supplementary Equation 10 |
| --- | --- |

## Rubber elasticity theory

In addition to swelling experiments, the $M_{c}$ was also estimated using the mechanical properties of equilibrium swollen samples. In their swollen state, most hydrogels exhibit a rubber-like mechanical behavior, with properties mainly related to the architecture of their polymer network (Anseth, Bowman, and Brannon-Peppas 1996). The general characteristics of rubber elastic behavior include high extensibility in response to low mechanical stress and complete recovery after load removal. From the constitutive relationships, the equilibrium shear modulus of the network derived from rubber elasticity model is given by Supplementary Equation 11 (Kennedy 1954):

| $G=\frac{\rho_{p}RT}{M_{c}}\left( 1-2\frac{M_{c}}{M_{n}} \right)$ | Supplementary Equation 11 |
| --- | --- |

where $G$ is the hydrogel shear modulus (in MPa), $\rho_{p}$ is the polymer density (in g/cm^3^), $R$ is the universal gas constant (i.e. 8.314472 J∙mol^-1^∙K^-1^) and $T$ is the absolute temperature (in K). It is worth noting that $M_{c}$ estimated using this approach is independent of the junction functionality. In agreement with the assumptions previously made, chain ends correction was neglected, obtaining the following expression for $M_{c}$:

| $\frac{1}{M_{c}}=\frac{G}{\rho_{p}RT}$ | Supplementary Equation 12 |
| --- | --- |

Usually, the equilibrium shear modulus ($G$) is taken equal to the plateau value of $G^{'}$ obtained from frequency sweep experiments performed on equilibrium swollen samples (i.e. $G_{s}^{'}$) (Lutolf and Hubbell 2003). In this work, since rheological measurements were performed in the hydrogel relaxed state, the $G_{s}^{'}$ was estimated from the measured $G_{r}^{'}$ according to Eq. S13. This expression was derived from the theory of rubber elasticity, which assumes that $G=const\cdot c^{1/3}$, where $c$ is the polymer volume fraction in a given state (i.e. $v_{2, r}$ or $v_{2,s}$ in case of relaxed or equilibrium swollen state, respectively) (Anseth, Bowman, and Brannon-Peppas 1996).

| $G_{s}^{'} =G_{r}^{'}\cdot\left( \frac{v_{2, r}}{v_{2, s}} \right)^{1/3}$ | Supplementary Equation 13 |
| --- | --- |

Alternatively, $G_{s}^{'}$ (which is almost equivalent to $G_{s}$ in case of materials with substantially elastic behaviour) can be derived from uniaxial compressive measurements on equilibrium swollen samples as the slope of the nominal stress versus $-\left( \lambda-\frac{1}{\lambda^{2}} \right)$, where $\lambda=l/l_{0}$ is the macroscopic deformation ratio, i.e. the ratio of the deformed ($l$) to the un-deformed ($l_{0}$) specimen length (Lin-Gibson et al. 2005; Beamish et al. 2010). The macroscopic deformation ratio can also be expressed as $\lambda=1-\varepsilon$, where $\varepsilon=(l_{0}-l)/l_{0}$ represents the compressive strain. The hydrogel average mesh size ($\xi$) was derived from the rubber elasticity theory using the following equation (Eq. S14) (Welzel et al. 2011; Lu and Anseth 2000; Cho 2016):

| $\xi=\left( \frac{G_{s}^{'}\cdot N_{A}}{RT} \right)^{-1/3}$ | Supplementary Equation 14 |
| --- | --- |

where $G_{s}^{'}$ (in Pa) is the hydrogel storage modulus in the equilibrium swollen state, $N_{A}$ the Avogadro’s constant (i.e. 6.02214129∙10^23^ mol^−1^), $R$ the universal gas constant (i.e. 8.314472 J∙mol^-1^∙K^-1^) and $T$ the absolute temperature (in K).

# Amine reaction characterization in soluble models and hydrogels

## Soluble models

Non-crosslinked precursor solutions of 0.75 A/DA hydrogels were used as representative soluble models to test both LOx and GlyO reactions with primary amines. Free amino groups (NH_2_) were measured over time using the Ninhydrin assay. When reacting with primary amines, the yellow Ninhydrin solution turns deep purple, with a maximum absorption at 570 nm (McCaldin 1960; Gebeyehu 2006). Please note that Eosin Y was not included in the soluble models to avoid any interference with spectrophotometric measurements. LOx and GlyO amino crosslinking solutions were prepared in 10 mM PBS at pH 7.4. The LOx crosslinking solution contains 0.1 unit/mL of enzyme supplemented with 5 μg/mL of L-ascorbic acid and 5 μg/mL of CuSO_4_ as enzyme cofactors (Elbjeirami et al. 2003; Rucker et al. 1998; Kuroyanagi et al. 2002; Murray and Levene 1977; Harris 1976). GlyO was stoichiometrically administered to have a 1:1 aldehyde:amine molar ratio. In particular, amine reactions were characterized by adding 700 μL of either LOx or GlyO amino crosslinking solution to 300 μL of hydrogel precursor solution in a 1.5 mL Eppendorf tube. Both LOx and GlyO reactions were carried out incubating the mixtures at 37 °C while gently shaking. Amino containing samples (i.e. 0.75-0.10 and 0.75-0.05 A/DA-NH_2_/A_PEGDA_) added with 700 μL of 10 mM PBS were used as negative controls for LOx and GlyO amine reactions. Conversely, 0.75-0.00 A/DA-NH_2_/A_PEGDA_ amine-free samples added with PBS, LOx or GlyO solutions were used as references for absorbance readings of amine-containing soluble models added with PBS, LOx or GlyO, respectively. The reaction kinetics were followed by sampling volumes of 200 μL from the incubated solution at 0, 0.5, 1, 3 and 18 h. Sampled volumes were transferred into 8 mL glass vials, topped with 200 μL of 2% w/v Ninhydrin reagent solution and gently mixed. Vials were placed in a boiling water bath for 10 minutes and then cooled to room temperature, according to the manufacturer’s instructions. Then, 5 mL of 95 % v/v ethanol were added to each vial and gently mixed. Finally, 200 μL volumes were transferred in a 96 well plate and the absorbance measured at 570 nm using a Biotek Synergy 2 plate reader (Biotek, Vermont, USA). Experiments were performed in triplicate.

A calibration curve (Supplementary Figure 2A) was obtained preparing standard samples in triplicate by consecutive dilutions of the 0.75-0.10 A/DA-NH_2_/A_PEGDA_ hydrogel precursor solution (containing 9.54 mM of NH_2_, Table 1) with an equal volume of amine-free 0.75-0.00 A/DA-NH_2_/A_PEGDA_ solution. The latter amine free solution was used as a reference for all absorbance readings. Standard samples were prepared as previously described.

## Hydrogel

The Ninhydrin assay protocol was adapted for crosslinked hydrogels according to the following procedure. First, the nine hydrogels were prepared in triplicate by photo-crosslinking 60 μL of the respective precursor solutions, as for swelling characterization. Since different hydrogels contain different amount of primary amines (please refer to A/DA-NH_2_/A_PEGDA_, Table 1), crosslinked hydrogels were placed in HPLC glass vials and used as standards to prepare the calibration curve (Supplementary Figure 2B). 140 μL of 10 mM PBS were added to the hydrogels in each vial such that the sample was complexly covered with the solution. An equal volume (200 μL) of Ninhydrin reagent 2 % w/v solution was added. Then, the vials were gently shaken, placed in boiling water bath for 10 minutes and cooled to room temperature. Subsequently, 1 mL of 95 % v/v ethanol was added and samples shaken for 5 minutes (500 rpm, room temperature). Finally, 400 μL of supernatant were collected from each vial and further diluted with an equal volume of 95 % v/v ethanol. Samples of 200 μL were transferred in a 96 well plate and the absorbance measured at 570 nm using a plate reader. Supernatants collected from vials containing amine-free hydrogels (i.e. 0.25-0.00, 0.50-0.00 and 0.75-0.00 A/DA-NH_2_/A_PEGDA_) were used as references for absorbance readings.

Two hydrogels were selected to characterize the amine reactions with LOx and GlyO, particularly the 0.75-0.10 A/DA-NH_2_/A_PEGDA_ hydrogels (characterized by the higher mesh size and amino content) and the 0.25-0.05 ones (exhibiting the lowest mesh size and amino content among those investigated). The LOx and GlyO amino crosslinking solutions were prepared in 10 mM PBS at pH 7.4 as previously described for soluble models. Briefly, 140 μL of amino crosslinking solution were added to 60 μL of photo-crosslinked hydrogel into HPLC glass vials. Amino containing hydrogels (i.e. 0.75-0.10 and 0.25-0.05 A/DA-NH_2_/A_PEGDA_) added with 140 μL of 10 mM PBS were used as negative controls for LOx and GlyO amine reactions, while 0.25-0.00 and 0.75-0.00 A/DA-NH_2_/A_PEGDA_ amine-free samples added with PBS, LOx or GlyO solutions were used as references for absorbance readings of amine containing hydrogels added with PBS, LOx or GlyO, respectively. Again, both LOx and GlyO reactions were carried out incubating vials at 37 °C while gently shaking. The amount of free amino groups was measured after 0, 1, 3, 18 and 48 h of reaction, processing samples as previously described. Experiments were performed in triplicate. Further analyses were performed administering glyoxal at 5:1 and 10:1 aldehyde:amine molar ratios to 0.25-0.05 A/DA-NH_2_/A_PEGDA_ hydrogels to better characterize GlyO amino reaction.

**
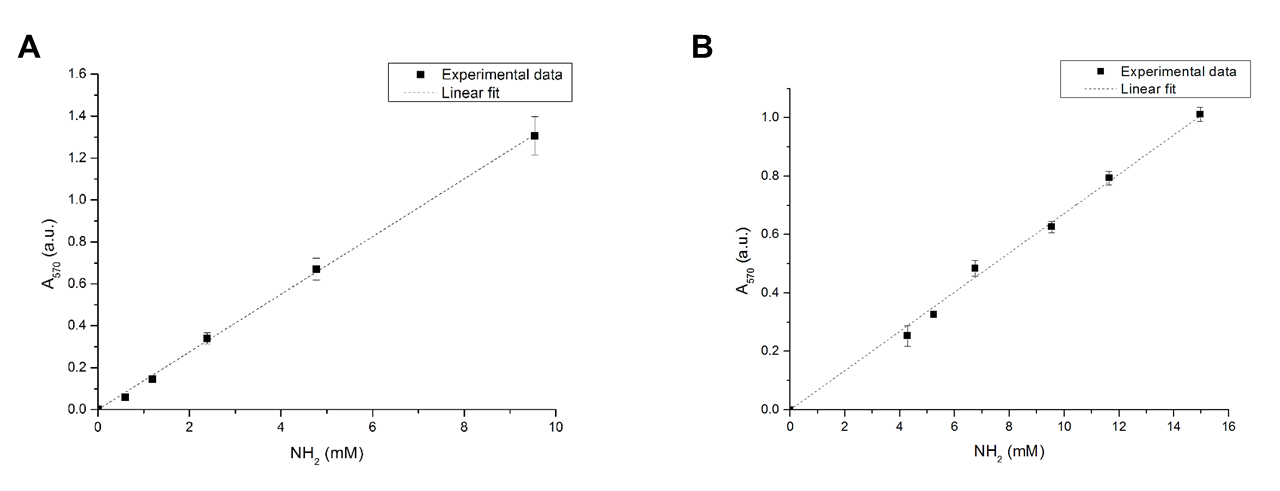
**

**Supplementary Figure 2.** Ninhydrin assay results. A) Calibration curve for primary amines in hydrogel soluble models. Absorbance measured at 570 nm increased linearly with NH_2_ concentration in the investigated range (*R^2^* = 0.999); B) Experimental calibration curve for primary amines obtained using photo-crosslinked hydrogels. Absorbance measured at 570 nm increased linearly with NH_2_ concentration (R^2^ = 0.998).

# CellTiter-Blue viability assay

CellTiter-Blue^®^ Cell Viability Assay (Promega, Mannheim, Germany) was used according to the manufacturer’s instructions to assess cell viability at day 1, 3 and 7. Briefly, the medium was replaced with 500 μL of fresh medium plus 100 μL of CellTiter-Blue^®^ Reagent and incubated for 150 minutes at 37 °C. Then, three 120 μL samples were transferred into a 96 well plate and analyzed for resofurin-mediated fluorescence (excitation, 544 nm; detection, 590 nm) in a microplate reader.

Hepatocyte metabolic function was investigated by measuring albumin and urea content in the culture media at different time points, i.e. day 1, 3 and 7.

Albumin was quantified using an enzyme-linked immunosorbent assay (ELISA) (Bethyl Laboratories Inc., Montgomery, TX) according to manufacturer’s instructions.

Urea content was measured using 10 μL of medium incubated with urease (Sigma-Aldrich) at 37 °C for 10 minutes. Then, phenol nitroprusside and alkaline hypochlorite solutions (Sigma-Aldrich) were added and urea content quantified after 20 minutes of incubation at room temperature by spectrophotometric readings at 660 nm using a FLUOstar Omega microplate reader (BMG Labtech GmbH, Offenburg, Germany) (Weatherburn 1967; CHANEY and MARBACH 1962).

# Live/Dead fluorescence viability testing

The Live/Dead viability assay (L3224; Invitrogen, Carlsbad, CA, USA) was used to evaluate hepatocyte viability at day 7. After removing the medium, hydrogels were washed twice with sterile PBS 1×. Samples were protected from the light and incubated with 500 μL of a sterile PBS 1× solution containing 4 μM ethidium homodimer-1 and 2 μM calcein-AM at 37 °C / 5 % CO_2_ for 30 minutes. After multiple washing with PBS 1× to remove Live/Dead reagents, samples were immediately analyzed with a confocal microscope (Nikon A1; Nikon Co. Ltd., Tokyo, Japan). The images acquired were then evaluated in terms of live (Ex/Em 494/517 nm) and dead (Ex/Em 536/617 nm) signals to estimate the mean percentage of viable cells with respect to the total number of encapsulated ones.

# Immunofluorescence staining

HepG2 cells were stained for F-actin and nuclei for further fluorescence observations of cell morphology at day 7. Samples were fixed in 4 % paraformaldehyde in Hank’s buffered salt solution (HBSS, Sigma) for 30 minutes at room temperature. Then, they were washed three times with PBS (Ca^2+^ and Mg^2+^ free). Formalin fixed cells were permeabilized with 0.1 % v/v Triton X-100 in PBS 1× for 5 minutes at room temperature and blocked with 5 % w/v bovine serum albumin (BSA; Sigma-Aldrich) in PBS for 30 minutes at room temperature. For F-actin filaments staining, samples were incubated in the dark with rhodamine-conjugated phalloidin (Life-Technologies) diluted 1:400 in PBS containing 1 % w/v BSA for 30 minutes at room temperature. After washing the samples three times with PBS, cell nuclei were stained incubating samples in the dark with 1 μg/mL DAPI (4’,6-diamidino-2 phenylindole, Sigma-Aldrich) solution in PBS 1× containing 1 % w/v BSA for 30 minutes at room temperature. Finally, samples were washed three times with PBS 1× and observed using a confocal microscope (Nikon A1, Nikon Co. Ltd., Tokyo, Japan).

# Assessment of hepatocyte metabolic function

Hepatocyte metabolic function was investigated by measuring albumin and urea content in the culture media at different time points, i.e. day 1, 3 and 7.

Albumin was quantified using an enzyme-linked immunosorbent assay (ELISA) (Bethyl Laboratories Inc., Montgomery, TX) according to manufacturer’s instructions.

Urea content was measured using 10 μL of medium incubated with urease (Sigma-Aldrich) at 37 °C for 10 minutes. Then, phenol nitroprusside and alkaline hypochlorite solutions (Sigma-Aldrich) were added and urea content quantified after 20 minutes of incubation at room temperature by spectrophotometric readings at 660 nm using a FLUOstar Omega microplate reader (BMG Labtech GmbH, Offenburg, Germany) (Weatherburn 1967; CHANEY and MARBACH 1962).

# Shear strain amplitude and frequency sweep

Typical shear strain amplitude and frequency sweep experimental results for a 0.50-0.10 A/DA-NH_2_/A_PEGDA_ hydrogel are shown in Supplementary Figure 3.


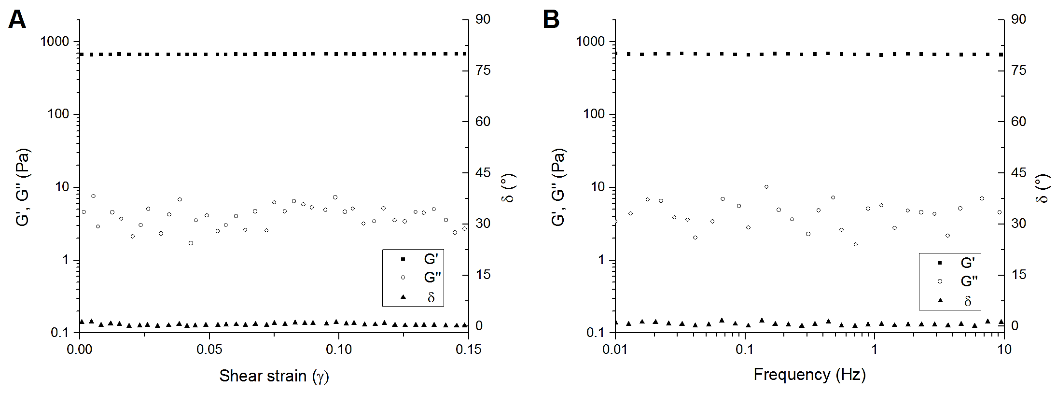


**Supplementary Figure 3.** Shear strain amplitude (A) and frequency sweep (B) results obtained for a 0.50-0.10 A/DA-NH_2_/A_PEGDA_ gel, showing the measured storage ($\boldsymbol{G}^{\boldsymbol{'}}$) and loss ($\boldsymbol{G}^{\boldsymbol{''}}$) shear moduli and the phase angle (δ).

# Creep-recovery

A typical plot of experimental creep-recovery angle over time is reported in Supplementary Figure 4, showing results obtained for a 0.50-0.10 A/DA-NH_2_/A_PEGDA_ gel.

After the application of the step of shear stress, samples reached immediately their equilibrium deformation, which remained constant over time during the entire creep phase. Notably, all samples completely recovered their deformation instantaneously when the stress was released, as expected for substantially elastic materials.


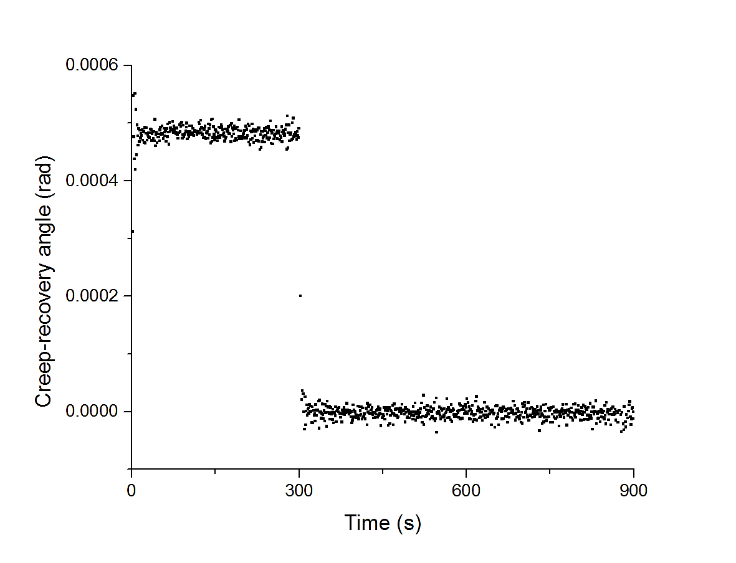


**Supplementary Figure 4.** Example of creep-recovery angle measured for a 0.50-0.10 A/DA-NH_2_/A_PEGDA_ gel, underlining the substantially elastic hydrogel mechanical behavior.

# Glyoxal amino crosslinking reaction in 0.25-0.05 A/DA-NH_2_/A_PEGDA_ gels at 5:1 and 10:1 aldehyde:amine molar ratios

These experiments were performed to investigate any concentration-related effect in the outcome of glyoxal (GlyO) amine reaction. It is worth noting that administering GlyO with aldehyde:amine molar ratio higher than 1 can possibly cause the quenching of free NH_2_, rather than the formation of new crosslinks within the hydrogel network (Martinez et al. 2007). Exploring this strategy is not of interest for the scope of the present work, but served only to better characterize the GlyO amine reaction. Amino content variations measured over time as a function of administered GlyO concentration are shown in Supplementary Figure 5 (samples added with PBS were used as controls).


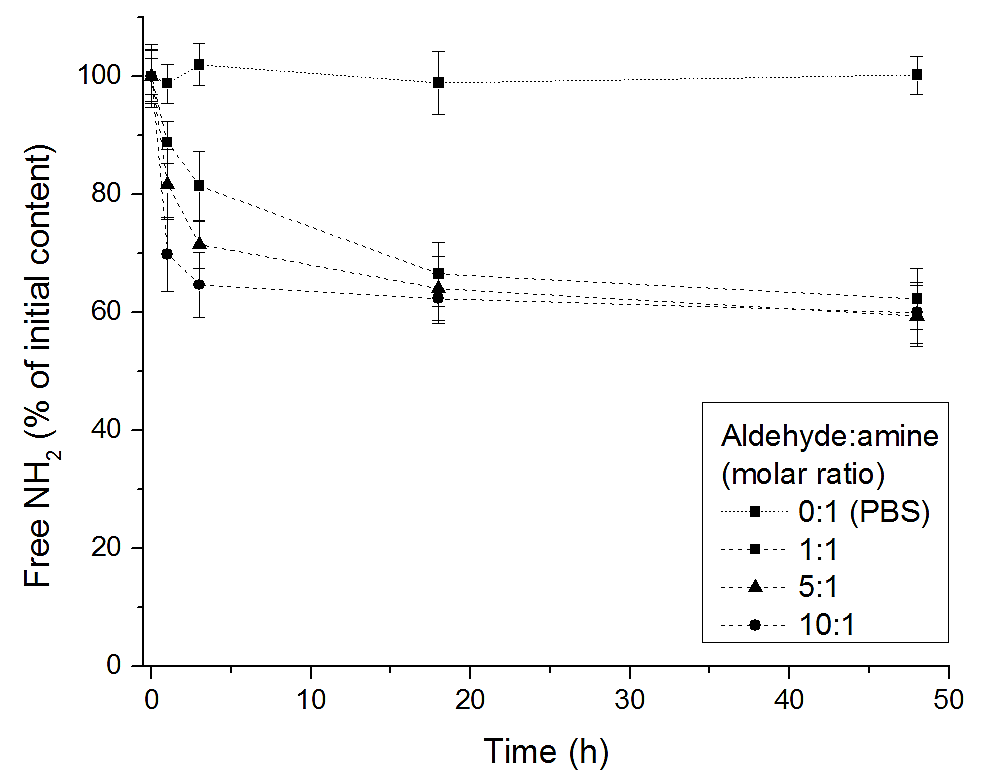


**Supplementary Figure 5.** Time variations of free amino content as a function of administered GlyO concentration (expressed as aldehyde:amine molar ratios) to 0.25-0.05 A/DA-NH_2_/A_PEGDA_ hydrogels. As expected, a faster reaction kinetics was observed with increasing GlyO concentration, whereas the plateau amine conversion did not change.

The increase of GlyO concentration returns faster reaction kinetics (one-way ANOVA followed by Tukey’s multiple comparisons test performed at each time point independently; *p* < 0.05 at both 1 and 3 hours). This result was expected since amine-glyoxal reaction is of the 1^st^ order with respect to both glyoxal and amine (total reaction order = 2) (De Haan, Tolbert, and Jimenez 2009; De Haan et al. 2009). Since the rate of GlyO amino crosslinking reaction is generally much higher than the diffusion rate of glyoxal molecule within hydrogels (i.e. diffusion-controlled kinetics) (Martinez et al. 2007), experimental concentration-dependent reaction kinetics suggest that the mesh sizes of investigated hydrogels allowed an effective diffusion of GlyO within hydrogel networks. The very low hydrodynamic diameter of this crosslinking agent (MW of GlyO is only 58.14 g/mol) and the $\xi$ estimations support this hypothesis. However, amine reaction conversion at plateau did not change significantly with glyoxal concentration (*p* > 0.05 at both 18 and 48 hours), indicating that the crosslinking has reached its maximum degree, with about 40 % of initial free NH_2_ conversion after 48 hours. These results are in agreement with Martinez et al., recently reporting on chitosan spheres crosslinked with glyoxal administered in various aldehyde:amine molar ratios (Martinez et al. 2007) and confirmed that GlyO administered in a 1:1 aldehyde:amine molar ratio can effectively be used as a positive control for LOx-mediated amino crosslinking reactions within investigated hydrogels.

# Calculated equilibrium swollen hydrogel shear moduli from compressive data

Compressive stress-strain ($\sigma$-$\varepsilon$) data within 0.15 strain obtained for all control samples incubated 48 hours in PBS 1× were used to derive SMS shear moduli in the equilibrium swollen state (i.e. $G_{s}\cong G_{s}^{'}$) as the slope of the nominal stress ($\sigma$) versus $-\left( \lambda-\frac{1}{\lambda^{2}} \right)$, where $\lambda=\frac{l}{l_{0}}=1-\varepsilon$, according to Lin-Gibson et al (Lin-Gibson et al. 2005). Supplementary Figure 6 shows $G_{s}^{'}$ values derived from compressive experiments in the equilibrium swollen state along with those estimated from shear measurements in the relaxed state using Eq. S13 (i.e. $G_{s}^{'}$ values used for mesh size estimations). No significant differences were found between hydrogels $G_{s}^{'}$ values estimated using the two different datasets, with results again dependent only on the A/DA molar ratio (one-way ANOVA, *p* < 0.05).


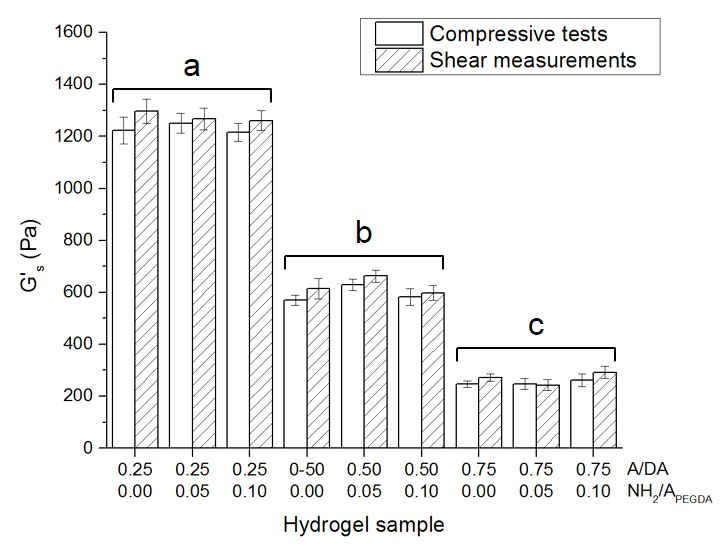


**Supplementary Figure 5.** Hydrogel shear storage moduli in the equilibrium swollen state ($\boldsymbol{G}_{\boldsymbol{s}}^{\boldsymbol{'}}$) estimated from compressive experiments in the equilibrium swollen state and from shear measurements in the relaxed state. Different letters indicate significant differences between samples (*p* < 0.05).

# References

Anseth, K S, C N Bowman, and L Brannon-Peppas. 1996. “Mechanical Properties of Hydrogels and Their Experimental Determination.” *Biomaterials* 17 (17): 1647–57. http://www.ncbi.nlm.nih.gov/pubmed/20473984.

Avens, Heather J., Erin L. Chang, Allison M. May, Brad J. Berron, Gregory J. Seedorf, Vivek Balasubramaniam, and Christopher N. Bowman. 2011. “Fluorescent Polymeric Nanocomposite Films Generated by Surface-Mediated Photoinitiation of Polymerization.” *Journal of Nanoparticle Research* 13 (1): 331–46. https://doi.org/10.1007/s11051-010-0034-z.

Beamish, Jeffrey A., Junmin Zhu, Kandice Kottke-Marchant, and Roger E. Marchant. 2010. “The Effects of Monoacrylated Poly(Ethylene Glycol) on the Properties of Poly(Ethylene Glycol) Diacrylate Hydrogels Used for Tissue Engineering.” *Journal of Biomedical Materials Research. Part A* 92 (2): 441–50. https://doi.org/10.1002/jbm.a.32353.

Canal, Tiziana, and Nikolaos A Peppas. 1989. “Correlation between Mesh Size and Equilibrium Degree of Swelling of Polymeric Networks.” *Journal of Biomedical Materials Research* 23 (10): 1183–93. https://doi.org/10.1002/jbm.820231007.

CHANEY, A. L., and E. P. MARBACH. 1962. “Modified Reagents for Determination of Urea and Ammonia.” *Clinical Chemistry* 8 (April): 130–32.

Cho, Kwang Soo. 2016. “Polymer Physics.” In *Springer Series in Materials Science*, 241:231–81. OUP Oxford. https://doi.org/10.1007/978-94-017-7564-9_4.

Elbjeirami, Wafa M, Edward O Yonter, Barry C Starcher, and Jennifer L West. 2003. “Enhancing Mechanical Properties of Tissue-Engineered Constructs via Lysyl Oxidase Crosslinking Activity.” *Journal of Biomedical Materials Research. Part A* 66 (3): 513–21. https://doi.org/10.1002/jbm.a.10021.

Ellman, George L., K. Diane Courtney, Valentino Andres, and Robert M. Featherstone. 1961. “A New and Rapid Colorimetric Determination of Acetylcholinesterase Activity.” *Biochemical Pharmacology* 7 (2): 88–95. https://doi.org/10.1016/0006-2952(61)90145-9.

Gebeyehu, Setegn. 2006. *Physiological Response to Drought Stress of Common Bean (Phaseolus Vulgaris L.) Genotypes Differing in Drought Resistance*. Cuvillier Verlag. https://books.google.com/books?id=remosd987CkC&pgis=1.

Haan, David O. De, Ashley L. Corrigan, Kyle W. Smith, Daniel R. Stroik, Jacob J. Turley, Frances E. Lee, Margaret A. Tolbert, Jose L. Jimenez, Kyle E. Cordova, and Grant R. Ferrell. 2009. “Secondary Organic Aerosol-Forming Reactions of Glyoxal with Amino Acids.” *Environmental Science and Technology* 43 (8): 2818–24. https://doi.org/10.1021/es803534f.

Haan, David O. De, Margaret a. Tolbert, and Jose L. Jimenez. 2009. “Atmospheric Condensed-Phase Reactions of Glyoxal with Methylamine.” *Geophysical Research Letters* 36 (11): L11819. https://doi.org/10.1029/2009GL037441.

Harris, E D. 1976. “Copper Induced Activation of Aortic Lysyl Oxidase in Vivo.” *Proceedings of the National Academy of Sciences of the United States of America* 73 (2): 371–74. https://doi.org/10.1073/pnas.73.2.371.

Jabbari, Esmaiel. 2011. “Bioconjugation of Hydrogels for Tissue Engineering.” *Current Opinion in Biotechnology*. Elsevier Ltd. https://doi.org/10.1016/j.copbio.2011.01.003.

Kennedy, Joseph W. 1954. “Principles of Polymer Chemistry.” *Journal of the American Chemical Society* 76 (10): 2854. https://doi.org/10.1021/ja01639a091.

Kuroyanagi, Miwa, Eriko Shimamura, Mihyan Kim, Nobuhiko Arakawa, Yoko Fujiwara, and Megumi Otsuka. 2002. “Effects of L-Ascorbic Acid on Lysyl Oxidase in the Formation of Collagen Cross-Links.” *Bioscience, Biotechnology and Biochemistry* 66 (10): 2077–82. https://doi.org/10.1271/bbb.66.2077.

Leach, Jennie Baier, Kathryn A Bivens, Charles W Patrick, and Christine E Schmidt. 2003. “Photocrosslinked Hyaluronic Acid Hydrogels: Natural, Biodegradable Tissue Engineering Scaffolds.” *Biotechnology and Bioengineering* 82 (5): 578–89. https://doi.org/10.1002/bit.10605.

Lin-Gibson, Sheng, Ronald L. Jones, Newell R. Washburn, and Ferenc Horkay. 2005. “Structure-Property Relationships of Photopolymerizable Poly(Ethylene Glycol) Dimethacrylate Hydrogels.” *Macromolecules* 38 (7): 2897–2902. https://doi.org/10.1021/ma0487002.

Lin, Haiqing, Teruhiko Kai, Benny D. Freeman, Sumod Kalakkunnath, and Douglass S. Kalika. 2005. “The Effect of Cross-Linking on Gas Permeability in Cross-Linked Poly(Ethylene Glycol Diacrylate).” *Macromolecules* 38 (20): 8381–93. https://doi.org/10.1021/ma0510136.

Lu, Sanxiu, and Kristi S. Anseth. 2000. “Release Behavior of High Molecular Weight Solutes from Poly(Ethylene Glycol)-Based Degradable Networks.” *Macromolecules* 33 (7): 2509–15. https://doi.org/10.1021/ma9915024.

Lutolf, M P, and J a Hubbell. 2003. “Synthesis and Physicochemical Characterization of End-Linked Poly(Ethylene Glycol)-Co-Peptide Hydrogels Formed by Michael-Type Addition.” *Biomacromolecules* 4 (3): 713–22. https://doi.org/10.1021/bm025744e.

Ma, Peter X, and Jennifer Elisseeff. 2005. *Scaffolding in Tissue Engineering*. *Scaffolding in Tissue Engineering*. CRC press. https://doi.org/10.1201/9781420027563.

Martinez, Leticia, Florence Agnely, Bernard Leclerc, Juergen Siepmann, Marine Cotte, Sandrine Geiger, and Guy Couarraze. 2007. “Cross-Linking of Chitosan and Chitosan/Poly(Ethylene Oxide) Beads: A Theoretical Treatment.” *European Journal of Pharmaceutics and Biopharmaceutics* 67 (2): 339–48. https://doi.org/10.1016/j.ejpb.2007.02.012.

McCaldin, D. J. 1960. “The Chemistry of Ninhydrin.” *Chemical Reviews* 60 (1): 39–51. https://doi.org/10.1021/cr60203a004.

Mellott, M B, K Searcy, and M V Pishko. 2001. “Release of Protein from Highly Cross-Linked Hydrogels of Poly(Ethylene Glycol) Diacrylate Fabricated by UV Polymerization.” *Biomaterials* 22 (9): 929–41.

Mezyk, Stephen P. 1995. “Rate Constant Determination for the Reaction of Sulfhydryl Species with the Hydrated Electron in Aqueous Solution.” *Journal of Physical Chemistry* 99 (38): 13970–75. https://doi.org/10.1021/j100038a031.

Murray, J C, and C I Levene. 1977. “Evidence for the Role of Vitamin B-6 as a Cofactor of Lysyl Oxidase.” *Biochemical Journal* 167 (2): 463–67. https://doi.org/10.1042/bj1670463.

Nair, Devatha P., Neil B. Cramer, John C. Gaipa, Matthew K. McBride, Emily M. Matherly, Robert R. McLeod, Robin Shandas, and Christopher N. Bowman. 2012. “Two-Stage Reactive Polymer Network Forming Systems.” *Advanced Functional Materials* 22 (7): 1502–10. https://doi.org/10.1002/adfm.201102742.

Park, Yong Doo, Nicola Tirelli, and Jeffrey a Hubbell. 2003. “Photopolymerized Hyaluronic Acid-Based Hydrogels and Interpenetrating Networks.” *Biomaterials* 24 (6): 893–900.

Peppas, N. a., Y. Huang, M. Torres-Lugo, J. H. Ward, and J. Zhang. 2000. “Physicochemical Foundations and Structural Design of Hydrogels in Medicine and Biology.” *Annual Review of Biomedical Engineering* 2 (1): 9–29. https://doi.org/10.1146/annurev.bioeng.2.1.9.

Rucker, Robert B, Taru Kosonen, Michael S Clegg, Alyson E Mitchell, Brian R Rucker, Janet Y Uriu-Hare, and Carl L Keen. 1998. “Copper, Lysyl Oxidase, and Extracellular Matrix Protein Cross-Linking.” *The American Journal of Clinical Nutrition* 67 (5 Suppl): 996S-1002S.

Rydholm, Amber E., Nicole L. Held, Danielle S.W. Benoit, Christopher N. Bowman, and Kristi S. Anseth. 2008. “Modifying Network Chemistry in Thiol-Acrylate Photopolymers through Postpolymerization Functionalization to Control Cell-Material Interactions.” *Journal of Biomedical Materials Research - Part A* 86 (1): 23–30. https://doi.org/10.1002/jbm.a.31526.

Weatherburn, M. W. 1967. “Phenol-Hypochlorite Reaction for Determination of Ammonia.” *Analytical Chemistry* 39 (8): 971–74. https://doi.org/10.1021/ac60252a045.

Welzel, Petra Birgit, Silvana Prokoph, Andrea Zieris, Milauscha Grimmer, Stefan Zschoche, Uwe Freudenberg, and Carsten Werner. 2011. “Modulating Biofunctional StarPEG Heparin Hydrogels by Varying Size and Ratio of the Constituents.” *Polymers* 3 (1): 602–20. https://doi.org/10.3390/polym3010602.
